# Closed-Loop Valorization of Annatto Seed Waste into Biochar: A Sustainable Platform for Phosphorus Adsorption and Safe Nutrient Recycling in Agro-Industries

**DOI:** 10.3390/molecules30132842

**Published:** 2025-07-02

**Authors:** Diana Guaya, Camilo Piedra, Inmaculada Carmona

**Affiliations:** 1 Department of Chemistry, Universidad Técnica Particular de Loja, Loja 110107, Ecuador; 2 Chemical Engineering School, Universidad Técnica Particular de Loja, Loja 110107, Ecuador; bcpiedra@utpl.edu.ec; 3 EcoSs_Lab, Department of Biological Sciences, Universidad Técnica Particular de Loja, Loja 110107, Ecuador; mocarincu@gmail.com

**Keywords:** annatto, waste valorization, metal-modified biochar, nutrient recovery, circular economy, wastewater treatment

## Abstract

Valorizing agro-industrial waste into functional materials for environmental remediation and resource recovery is essential for advancing circular economy models. This study presents a novel closed-loop strategy to convert annatto (*Bixa orellana*) seed residues into biochar for phosphate recovery from aqueous solutions and real agro-industrial wastewater. A novel ternary modification with Fe, Zn, and Mn metals was applied to enhance the phosphate adsorption performance of the biochar. Materials were synthesized via pyrolysis at 600 °C and 700 °C, with ABC-M700 exhibiting the highest performance. Comprehensive characterization (FTIR, SEM–EDS, and XRF) confirmed the successful incorporation of metal (oxy)hydroxide functional groups, which facilitated phosphate binding. Adsorption studies revealed that ABC-M700 achieved a maximum phosphate removal capacity of 6.19 mg·g^−1^, representing a 955% increase compared to unmodified ABC-N700 (0.59 mg·g^−1^), and a 31% increase relative to ABC-M600 (4.73 mg·g^−1^). Physicochemical characterization indicated increased surface area, well-developed mesoporosity, and the formation of metal (oxy)hydroxide functionalities. ABC-M700 achieved a maximum adsorption capacity of 73.22 mg·g^−1^ and rapid kinetics, removing 95% of phosphate within 10 min and reaching equilibrium at 30 min. The material exhibited notable pH flexibility, with optimal performance in the range of pH 6–7. Performance evaluations using real wastewater from the same agro-industry confirmed its high selectivity, achieving 80% phosphate removal efficiency despite the presence of competing ions and organic matter. Phosphate fractionation revealed that 78% of adsorbed phosphate was retained in stable, metal-associated fractions. Although the material showed limited reusability, it holds potential for integration into nutrient recycling strategies as a slow-release fertilizer. These findings demonstrate a low-cost, waste-derived adsorbent with strong implications for circular economy applications and sustainable agro-industrial wastewater treatment. This study establishes a scalable model for agro-industries that not only reduces environmental impact but also addresses phosphorus scarcity and promotes resource-efficient waste management.

## 1. Introduction

The generation and disposal of agro-industrial waste represents a major environmental challenge, particularly in the food processing sector. The growing global demand for sustainable waste management practices has made this issue increasingly urgent [1]. One of the most promising approaches to address agro-industrial waste is its valorization through the production of biochar, a carbon-rich material obtained via pyrolysis of organic matter [2]. Biochar has gained wide recognition due to its diverse applications in carbon sequestration, soil remediation, and water treatment [3].

The production of biochar from agricultural residues has been extensively studied, with numerous reports highlighting its benefits in soil health, carbon storage, and pollutant adsorption [4]. Biochar improves soil structure, water retention, and nutrient availability by effectively adsorbing nutrients such as nitrogen and phosphorus, thereby preventing their loss to the environment [5]. Although traditional feedstocks such as corn husks, rice straw, and wood chips have been well documented, less attention has been given to other abundant agro-industrial residues [6]. Recent studies have explored unconventional waste sources, such as fruit peels, coffee grounds, and other food industry-derived by-products, which have shown promising adsorption properties for heavy metals and nutrients [7].

Among such underexplored residues is annatto (*Bixa orellana*), a plant widely used as a natural colorant in the food and cosmetic industries [8], generating large quantities of residual biomass typically discarded or minimally used for composting [9]. Despite the broad literature on biochar for water treatment [10], research on biochar derived specifically from annatto residues remains scarce. To our knowledge, no previous studies have reported the synthesis or application of annatto seed-derived biochar for pollutant removal [11]. Considering the large volume of waste generated from annatto processing in condiment production facilities, transforming this waste into functional biochar presents an innovative and sustainable approach to wastewater treatment within the same industry [12]. This underutilized residue holds substantial potential for phosphorus–phosphate recovery from wastewater and subsequent application in agriculture to enhance soil fertility. Phosphorus, an essential macronutrient in agriculture, is often lost through overapplication and leaching, leading to environmental problems such as eutrophication and reduced soil fertility [13]. Recovering phosphorus from wastewater and reintroducing it into soil systems in a slow-release form offers both environmental and agronomic advantages [14]. Given that phosphorus is a non-renewable resource and that global reserves are depleting, its recovery from waste streams has become increasingly necessary [15].

While traditional biochar can contribute to soil improvement, their unmodified forms often show limited capacity for effective phosphate adsorption and controlled release [16]. A promising strategy involves aqueous-phase impregnation of metals such as Fe, Mn, or Zn without thermal activation, yielding surface-bound metal (oxy)hydroxide groups with a high affinity for phosphate via mechanisms including ligand exchange and electrostatic attraction [17,18]. However, studies incorporating Mn/Zn/Fe (oxy)hydroxides onto biochar are still limited. In contrast to our previous work, where Mn/Zn/Fe (oxy)hydroxides were synthesized on a synthetic Mg–Al LDH matrix under tightly controlled pH and temperature conditions using high-purity reagents [18], the current work utilizes an underutilized and abundant agro-industrial by-product both as a carbon precursor and a reactive substrate. Moreover, by incorporating soil-compatible micronutrients such as Fe, Zn, and Mn, the resulting material not only effectively removes phosphate but also serves as a potential slow-release fertilizer, supporting sustainable agricultural reuse [18].

To the best of our knowledge, this is the first report on the use of biochar synthesized from annatto (*Bixa orellana*) seed waste for phosphate recovery from wastewater. This valorization pathway not only addresses waste generated from annatto-based processing but also provides a practical model for integrating the circular economy into economic systems. By modifying the biochar with micronutrients essential for plant growth, this work simultaneously addresses the challenges of wastewater decontamination and nutrient recycling [19]. The process represents a novel closed-loop valorization strategy: annatto waste from a local food-processing facility is converted into a ternary metal-modified biochar, which is then applied to treat wastewater from the same facility for phosphate removal and nutrient recycling in agriculture.

The objective of this study was to evaluate the feasibility and performance of metal-modified annatto biochar in removing phosphate from real agro-industrial wastewater. Specifically, the goals were as follows: (i) to synthesize biochar from annatto seed waste; (ii) to chemically modify the biochar with Fe, Zn, and Mn to enhance phosphate adsorption; (iii) to characterize the materials’ physicochemical properties; (iv) to evaluate phosphate adsorption behavior under varying pH, kinetic, and isotherm conditions; (v) to assess phosphate recovery through fractionation and desorption studies; and (vi) to validate phosphate removal performance in real wastewater from the annatto industry. This work contributes to sustainable waste management and soil science by offering practical solutions for phosphorus recovery and reuse. It also lays the foundation for future research on reintroducing phosphorus to the soil in slow-release forms, thereby maintaining soil fertility and minimizing the environmental impact of phosphate fertilizers.

## 2. Results and Discussion

### 2.1. Effect of Calcination and Temperature on the Adsorption Capacity of Annatto Biochar

**Figure 1:**
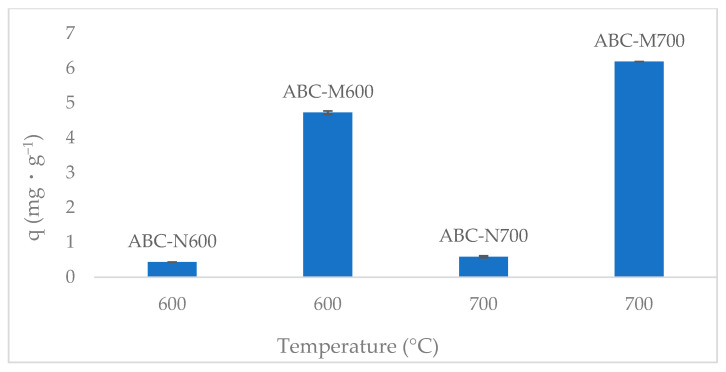
Phosphate adsorption capacity (q) of biochar derived from annatto seed waste, calcined at 600 °C and 700 °C in its natural forms (ABC-N600, ABC-N700) and metal-modified forms (ABC-M600 and ABC-M700).

Figure 1 presents the phosphate adsorption capacity of ABC-N600, ABC-N700, ABC-M600, and ABC-M700 as a function of calcination temperature. An increase in phosphate adsorption capacity was observed from 0.44 mg·g^−1^ for ABC-N600 to 0.59 mg·g^−1^ for ABC-N700, representing a 35% improvement. This enhancement is consistent with trends commonly reported in the biochar literature regarding thermal treatment. Although comparative analysis of surface area, FTIR, or elemental composition was not specifically performed for these samples, two widely recognized phenomena may explain the observed increase. First, pyrolysis temperature plays a critical role in modifying the physicochemical properties of biochar, including surface area, pore volume, and functional group composition, all of which directly influence adsorption performance [20,21]. Elevated pyrolysis temperatures typically promote the development of micropores and mesopores, providing more diffusion pathways for phosphate ions and enhancing adsorption efficiency [22]. Second, higher thermal treatment leads to a reduction in oxygen-containing functional groups such as −COOH and −OH [23,24]. These groups may otherwise compete with phosphate ions for active sites on the biochar surface due to competitive interactions [25], thereby decreasing adsorption efficiency at lower pyrolysis temperatures. The combined effect of increased porosity and reduced competition from oxygenated groups contributes to the formation of a cleaner, more stable surface structure with a greater affinity for phosphate ions.

A substantial enhancement in phosphate adsorption capacity was observed following metal modification. ABC-M600 exhibited a capacity of 4.73 mg·g^−1^, representing a 987% increase compared to its unmodified counterpart (ABC-N600). Similarly, ABC-M700 reached 6.19 mg·g^−1^, corresponding to a 955% increase relative to ABC-N700 and a 31% improvement over ABC-M600. These findings indicate that while pyrolysis temperature has a moderate influence on phosphate uptake, the incorporation of Fe, Zn, and Mn (oxy)hydroxide functional groups is the primary factor driving adsorption performance. This substantial improvement is attributed to the presence of metals that enhance surface reactivity and introduce additional active binding sites for phosphate. These are formed through the development of metal (oxy)hydroxide groups that improve the adsorption capacity of the biochar matrix [26]. Previous studies on metal-modified adsorbents have shown that metal ions readily form complexes with phosphate, leading to stronger and more selective adsorption interactions [18]. The superior performance of ABC-M700 reached 6.19 mg·g^−1^, surpassing ABC-N700 by 955% and ABC-M600 by 31%, can also be attributed to structural changes induced by higher pyrolysis temperatures, which facilitate more efficient adsorption. Elevated temperatures promote matrix restructuring and the formation of microstructures, which facilitate a more uniform distribution of metal (oxy)hydroxide compounds within the porous framework. These outcomes align with established adsorption mechanisms such as complexation, chelation, ion exchange, and surface precipitation, which are commonly observed in metal-doped biochar [27]. Comparable findings have been reported for biochar derived from other feedstocks, emphasizing the importance of optimizing both the thermal calcination and metal impregnation stages to enhance adsorption performance [28]. Nonetheless, the dominant contribution to phosphate uptake in this case is attributed to the modified surface chemistry, particularly the formation of specific active sites that favor chemisorption and surface complexation with phosphate, far surpassing improvements from pyrolysis temperature alone. Based on its superior phosphate adsorption capacity, the metal-modified biochar calcined at 700 °C (ABC-M700) was selected for subsequent analysis. To simplify the presentation and ensure consistency, all results and discussions hereafter refer exclusively to ABC-M700.

### 2.2. Biochar Physicochemical Characterization

Images of the raw annatto seed residues before pyrolysis and the metal-modified biochar (ABC-M700) are provided in the Supplementary Materials (Figure S1). The visual comparison between raw annatto seed waste (reddish-orange, fibrous) and the resulting biochar (black, powder) obtained after pyrolysis at 700 °C illustrates the structural and color transformation due to carbonization. The elemental compositions of unmodified biochar (ABC-N700) and modified biochar (ABC-M700) are summarized in Table 1. The major elements in ABC-N700 include MgO, Al_2_O_3_, SiO_2_, P_2_O_5_, S, K_2_O, CaO, and Fe_2_O_3_, while minor elements such as Mn, Zn, and Ba were also detected. Although XRF analysis has limitations in detecting elements with atomic numbers below 23 (e.g., carbon and nitrogen), the high potassium and silica contents are characteristic of biochar derived from plant materials rich in mineral ash, which enhances their base-catalytic potential in environmental applications. In ABC-M700, increases in Mn (0.24%), Fe_2_O_3_ (0.52%), and Zn (0.71%) confirmed the efficacy of the modification process, during which manganese (Mn), iron (Fe), and zinc (Zn) were successfully incorporated into the biochar matrix. Simultaneously, a significant decrease in MgO (from 3.26 ± 0.48% to 0.91 ± 0.05%) and K_2_O (from 11.10 ± 0.05% to 2.81 ± 0.03%) was observed, while CaO increased from 2.63 ± 0.02% to 3.60 ± 0.02%. The increased CaO content in ABC-M700 may be attributed to the enhanced stabilization of the biochar matrix during the modification process [29]. The reductions in MgO and K_2_O suggest that an ion exchange process occurred during modification, in which native cations such as Mg^2+^ and K^+^ were replaced by the introduced Zn^2+^, Mn^2+^, and Fe^3+^ ions due to their higher binding affinity for the functional groups on the biochar, allowing the original cations to be released into the modifying solution [30]. Additionally, mechanisms such as surface adsorption, precipitation, diffusion, complexation, and chelation contribute to the incorporation of these metal ions [31]. The higher concentration of iron oxide in ABC-M700 suggests the formation of Fe oxide species, which, together with Zn and Mn oxide species, may be deposited on the biochar surface, as reported for other metal-modified and calcined biochar [32,33]. Overall, modification with transition metals is known to enhance the physicochemical properties of biochar (e.g., surface area and adsorption affinity) more effectively than modification with non-metals or alkali/alkaline earth metals [34].

**Figure 2:**
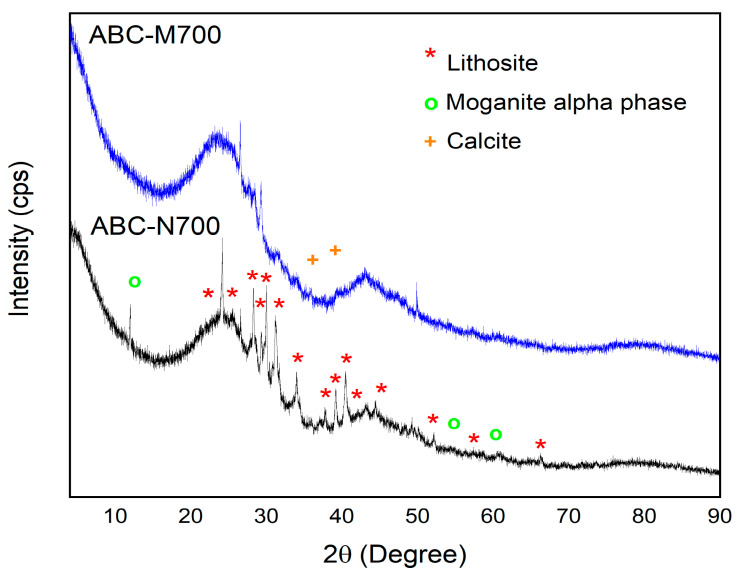
XRD patterns of natural (ABC-N700) and metal-modified annatto biochar (ABC-M700).

The x-ray diffraction (XRD) patterns of) both the unmodified (ABC-N700and metal-modified (ABC-M700) biochar are presented in Figure 2. Broad peaks, especially in the 20–30° (2θ) range, suggest the presence of amorphous carbon, a characteristic feature of biochar resulting from incomplete carbonization [35]. The diffraction peak near 30° (2θ) is commonly associated with silicates or other mineral phases often retained in biochar derived from plant-based precursors [36]. The principal diffraction peaks of ABC-N700 were compared against standard patterns available in the Crystallography Open Database [37]. Two primary mineral phases were identified in ABC-N700: lithosite (Ref. 9011907) and moganite alpha phase (Ref. 1550017). Lithosite exhibited reflections at 2θ values of 23.5°, 25.4°, 26.0°, 28.3°, 29.6°, 30.0°, 31.2°, 31.7°, 34.0°, 38.0°, 39.2°, 40.4°, 43.0°, 45.9°, 52.0°, 60.4° and 66.3°, and was indexed to a monoclinic crystal system (space group P 1 21 1) with refined unit cell parameters: a[Å] = 8.43, b[Å] = 10.35 and c[Å] = 15.14. The moganite alpha phase displayed diffraction peaks at 2θ values of 11.0°, 54.0° and 58.9°, indexed to an orthorhombic crystal system (space group P c c m), with refined unit cell parameters: a[Å] = 4.89, b[Å] = 10.98, and c[Å] = 9.07. In ABC-M700, slight shifts in peak intensity and position were observed, attributed to changes in the crystallographic environment caused by metal incorporation. Notably, new diffraction peaks at 2θ values of 36.0° and 39.7° were attributed to the presence of calcite (Ref. 1547347). Similar diffraction patterns were reported by Profeta et al. (2025) and Faria et al. (2024), who likewise identified calcite in carbonaceous and zeolitic composite materials [38,39]. The appearance of the calcite phase is likely due to a secondary mineralization process, resulting from CO_2_ interactions during pyrolysis or subsequent environmental exposure. Calcite, which may form through carbonation reactions or interactions with residual alkali or alkaline earth metals, underscores the propensity of biochar matrices to undergo secondary mineral transformations after pyrolysis [40]. Moreover, interactions between calcium and phosphate ions can result in the formation of calcium compounds, thereby reinforcing the role of biochar in nutrient recovery applications [41]. Calcite is indexed to the trigonal crystal system (space group R -3 c) with unit cell parameters a[Å] = b[Å]: 4.99 and c[Å] = 10.05. The parent sample, ABC-N700, exhibited a d-spacing of 3.15 Å at the diffraction peak (2θ ≈ 28°), corresponding to the hkl (032) reflection, which contracted to 3.11 Å in ABC-M700. This shift suggests a slight structural rearrangement and modification of the lithosite lattice parameters, potentially attributable to the substitution of native lattice cations by Zn^2+^, Mn^2+^, and Fe^3+^ ions. Furthermore, the increased “noise” observed in the diffractograms of ABC-M700 supports the hypothesis that amorphous or non-crystalline metal phases have formed, as no additional crystalline phases were detected. The incorporation of these transition metals induces lattice strain and distortions without disrupting the overall mineral framework, resulting in observable changes in peak intensities and a reduction in interplanar spacing [18]. The co-precipitation of iron, manganese, and zinc hydroxide particles, Fe(OH)_3_(s), Zn(OH)_2_(s), and Mn(OH)_2_(s), on the biochar surface was facilitated by adjusting the pH to 9 using NaOH. This adjustment promoted the nucleation and deposition of metal (oxy)hydroxide phases within the porous structure, as corroborated by the elemental composition and further confirmed by SEM analysis. According to the Medusa speciation diagrams (Figure S2) provided in the Supplementary Materials, the aqueous speciation of Fe^3+^, Zn^2+^, and Mn^2+^ at 25 °C confirms the formation domains of metal (oxy)hydroxides, specifically Fe(OH)_3_, Mn(OH)_2_, and Zn(OH)_2_, under the conditions used for biochar modification. This results in a mixture of aqueous species [M^n+^(aq)] and OH^−1^ (aq)], as well as solid-phase species [M(OH)_n_(s)] [17].

**Figure 3:**
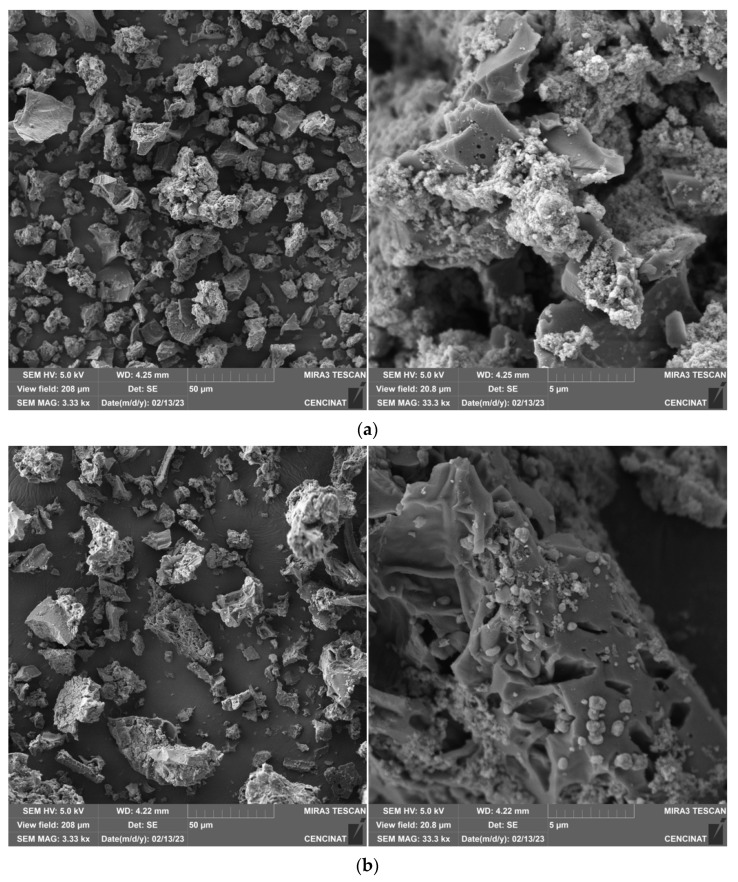
SEM image of (**a**) natural (ABC-N700) and (**b**) metal-modified annatto biochar (ABC-M700) at 50 µm and 5 µm scales.

SEM micrographs of the annatto biochar are presented in Figure 3a,b, illustrating the distinct morphological characteristics of the pristine material and the structural changes induced by the incorporation of metal species (Zn, Mn, Fe). In the unmodified biochar ABC-N700 (Figure 3a), low-magnification images reveal a compact, granular morphology with an irregular, fragmented structure and a heterogeneous particle size distribution. The surface displays a pronounced rough texture, interspersed with micro-fractures, which are indicative of thermal degradation of lignocellulosic components during pyrolysis [4]. These morphological features are consistent with those typically observed in biochar derived from similar biomass sources. At higher magnification, a more complex and disordered porous network is evident, characterized by significant surface roughness and voids dispersed throughout the carbon matrix. This porosity is characteristic of lignocellulosic biomass pyrolyzed at moderate to high temperatures and depends on the experimental conditions used during biochar production. Volatilization of organic compounds during pyrolysis results in a carbon-rich matrix containing numerous voids. Studies have shown that biochar produced at elevated temperatures, such as 700 °C, typically exhibits enhanced porosity and surface area [42]. Conventionally, the original cell wall architecture of plants is disrupted, leading to a disordered structure. In the modified biochar (ABC-M700, Figure 3b), the surface exhibits significant morphological changes, characterized by the presence of numerous small particles distributed across the surface. These features can be attributed to the formation of a solid carbon structure during pyrolysis, followed by interactions between volatile gases and the carbon matrix, leading to the generation of small-molecule gases and ash deposits that accumulate within the pore channels, as similarly observed in corn-based biochar [42]. ABC-M700 shows pronounced granulations and agglomerates compared to ABC-N700, suggesting the precipitation of metal oxides and hydroxides within the biochar matrix and its pore network [18]. These morphological transformations observed via SEM are consistent with findings from other studies reporting enhanced structural and functional properties in metal-impregnated biochar [31,33,43]. Furthermore, the presence of metal-rich nodules contributes to increased surface roughness, resulting in a more heterogeneous surface texture. However, the modification process appears to partially obstruct pore channels, as indicated by smoother and more compact regions visible in the SEM images. This effect may result from metal impregnation either filling existing voids or forming new surface layers, thereby altering the porosity of the biochar. BET analysis indicated a significant increase in specific surface area from 4 m^2^·g^−1^ (ABC-N700) to 33 m^2^·g^−1^ (ABC-M700). This suggests that the introduction of metal oxides, such as zinc, iron, and manganese, further influences porosity by generating additional microstructures or altering the existing pore architecture. Multiple studies have demonstrated that metal impregnation enhances both surface area and porosity, thereby significantly improving the adsorption performance of biochar [44,45].

**Figure 4:**
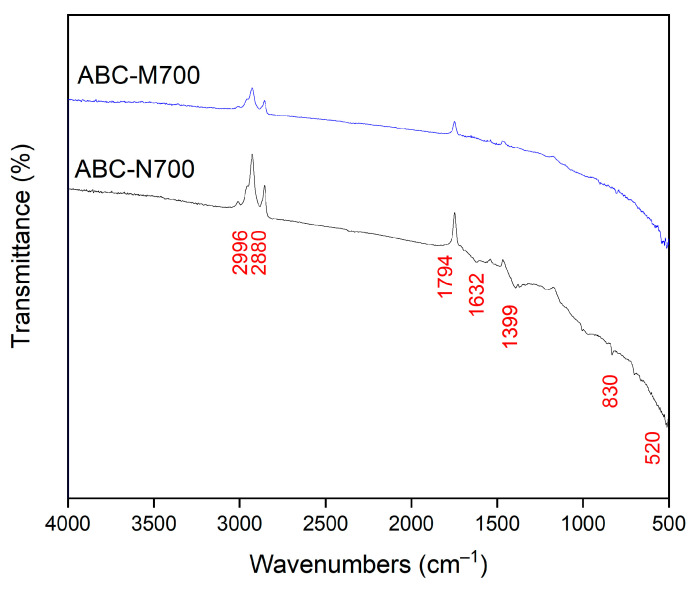
FTIR spectrum of natural ABC-N700 and modified annatto biochar ABC-M700.

The Fourier Transform Infrared (FTIR) spectra of both the unmodified annatto biochar (ABC-N700) and its metal-modified form (ABC-M700) are shown in Figure 4. The main wavenumber positions identified were 2996, 2880, 1794, 1632, 1399, 830, and 520 cm^−1^. The prominent absorption bands in ABC-N700 at 2996 cm^−1^ and 2880 cm^−1^ are attributed to the C-H stretching vibrations of aliphatic methylene groups (-CH_2_-) present in the biochar structure [44,46]. These peaks indicate the presence of saturated hydrocarbons and are characteristic of the lignocellulosic origin of the biochar. The band near 1794 cm^−1^ corresponds to the C=O stretching vibrations of carbonyl groups (e.g., ketones, aldehydes, and carboxylic acids). The peak at 1632 cm^−1^ is indicative of C=C stretching vibrations in aromatic rings, reflecting the degree of aromaticity and graphitization within the biochar [46]. The 1399 cm^−1^ peak is associated with C-H bending vibrations in methyl and methylene groups, further corroborating the presence of aliphatic structures [33]. The absorption band at 830 cm^−1^ is attributed to out-of-plane C-H bending vibrations in aromatic systems [42]. The FTIR spectrum of ABC-M700 shows modifications in both peak intensity and position, particularly at 2996, 2880, 1794, 1632, 1399, and 830 cm^−1^. These peaks remain present but display reduced intensity and a slight shift to lower wavenumbers, indicating alterations in the local chemical environment due to the incorporation of Zn, Mn, and Fe. The observed changes in the aliphatic chains may result in the formation of new bonding environments or the disruption of existing structures [42]. The persistence of carbonyl functionalities after modification can be attributed to the formation of metal–carboxylate complexes [47]. Aromatic C=C structures also appear to be affected, likely due to changes in electron density and aromaticity introduced by metal doping [48]. Furthermore, the attenuation of the aliphatic C-H bending bands may result from the development of more stable aromatic structures at metal-functionalized sites [49]. In the lower wavenumber region 500–830 cm^−1^, characteristic metal–oxygen (M-O) stretching vibrations are expected. The FTIR spectrum of ABC-M700 exhibited a distinct band at approximately 520 cm^−1^, which was significantly weaker in the unmodified biochar. This band is attributed to M-O stretching vibrations and serves as spectroscopic evidence for the formation of surface-bound metal (oxy)hydroxides (Fe-O, Mn-O, Zn-O) on the modified biochar surface [50,51]. These findings are consistent with previous studies on metal-impregnated sorbents synthesized via aqueous co-precipitation under ambient conditions [13,17,18].

### 2.3. Uptake of Phosphate as a Function of pH

**Figure 5:**
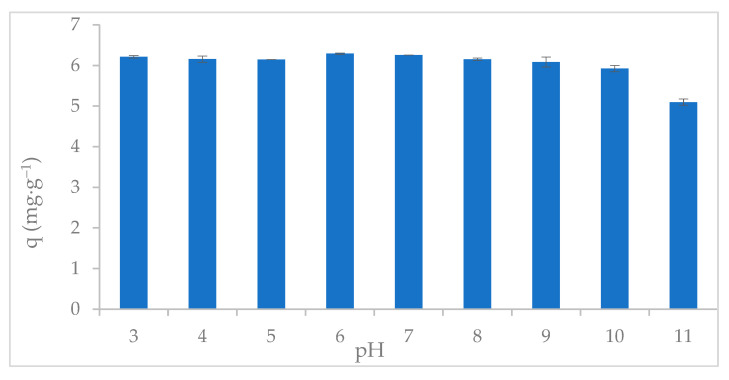
Phosphate adsorption capacity (q) of metal-modified annatto biochar (ABC-M700) as a function of pH.

The pH-dependent adsorption behavior of ABC-M700 showed high phosphate uptake efficiency (>85%) over a broad pH range. ABC-M700 demonstrated wide applicability across various water conditions, with optimal phosphate uptake observed between pH 6 and 7 (Figure 5), where the adsorption capacities were approximately 6.29 and 6.26 mg PO_4_^3−^∙g^−1^, respectively. This pH range aligns well with the typical pH levels found in wastewater systems, enabling the direct application of biochar without extensive pH adjustments. The point of zero charge (pH_PZC_) was determined to be 9.85 ± 0.1, at which the biochar surface exhibited no net charge, thereby promoting a balanced adsorption of both anionic and cationic species. This characteristic is particularly advantageous for phosphate removal in wastewater systems with a near-neutral pH (ranging from 7 to 9.85 ± 0.1), as it maximizes the interaction between phosphate anions and the positively charged sites on the biochar. These interactions reflect the dominance of metal oxyhydroxide functional groups (-Fe-OH, -Zn-OH, -Mn-OH) introduced during chemical modification, which can undergo protonation and deprotonation in response to the solution pH. Upon protonation, these surface hydroxyl groups form positively charged sites -M-OH_2_^+^ (where M = Fe, Zn, Mn), enabling the strong electrostatic attraction of anionic phosphate species (H_2_PO_4_^−^/HPO_4_^2−^), thereby enhancing phosphate adsorption below pH 9.85, as described by Equation (1) [52].

(1) $$ABC-M700-OH\begin{array}{c} \overset{Protonation}{\to }\\ pH<pH_{PZC} \end{array}ABC-M700-OH^{2+}\begin{array}{c} \begin{array}{c} Electrostatic\\ attraction \end{array}\\ \underset{H_{2}PO_{4}^{-}/HPO_{4}^{2-}}{\to } \end{array}\begin{array}{c} \begin{array}{c} ABC-M700-OH^{2+}\ldots H_{2}PO_{4}^{-} \end{array}\\ ABC-M700-OH^{2+}\ldots HPO_{4}^{2-} \end{array}$$

Above pH 9.85 ± 0.1, surface deprotonation generates negatively charged sites (-M-O^−^) on the biochar, which repel phosphate anions and reduce adsorption, as indicated by Equation (2). At basic pH (pKa_3_ = 12.36), further increases in pH lead to the gradual conversion of HPO_4_^2−^ to PO_4_^3−^, resulting in a reduction in phosphate removal. This reduction is attributed to both the high negative charge (–3) of the PO_4_^3−^ ion and the formation of the hydrated, negatively charged hydroxide surface (ABC-M700-MO^−^) [53].

(2) $$ABC-M700-OH\begin{array}{c} \overset{Hydroxilation}{\to }\\ pH\gg pH_{PZC} \end{array}ABC-M700-O^{-}+H_{2}O$$

Additionally, the pore size distribution in ABC-M700, consisting of micropores (<2 nm) and mesopores (2–50 nm), with a specific surface area of 33 m^2^·g^−1^, facilitates the physical adsorption of phosphate. Moreover, cations (K^+^, Ca^2+^, Mg^2+^) present in ABC-M700 can electrostatically bind phosphate ions (H_2_PO_4_^−^ and HPO_4_^2−^) to the negatively charged biochar surfaces at near-neutral pH. The literature suggests that biochar with high surface area and microporosity, such as annatto biochar, can exhibit strong adsorption performance for water contaminants through both physical adsorption and electrostatic interactions.

### 2.4. Adsorption Kinetics

**Figure 6:**
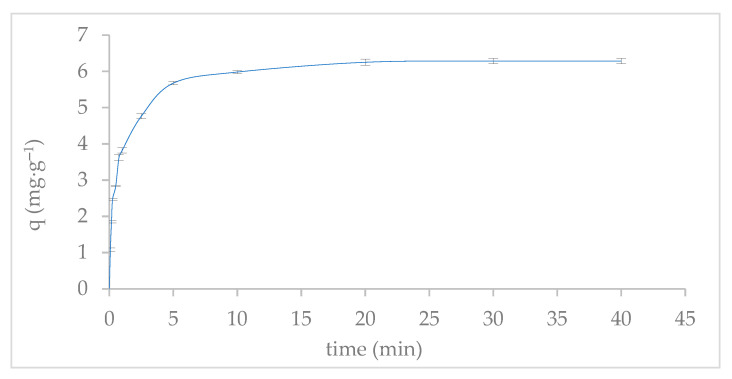
Phosphate adsorption kinetics of metal-modified annatto biochar (ABC-M700). Experimental data are presented as mean ± standard deviation from triplicate measurements.

The kinetics of phosphate adsorption onto ABC-M700 revealed rapid uptake, with approximately 95% of the phosphate ions adsorbed within the first 10 min, and equilibrium was reached within 30 min (Figure 6). However, for the kinetic modeling, the adsorption data were first averaged and then fitted using nonlinear regression. As such, the kinetic parameters reported in Table 2 correspond to the best-fit values derived from the mean dataset, and no individual replicate modeling was performed. This approach ensures consistency in parameter estimation, although standard deviations for the parameters are not provided in the table. These rapid kinetics support the suitability of ABC-M700 for real-time wastewater treatment, where prompt phosphate removal is critical.

The experimental data were analyzed to elucidate the underlying adsorption mechanisms (Table 2). The values presented represent the best-fit parameters obtained from averaged triplicate data; standard deviations are not reported as the fitting was performed on mean values only. The adsorption kinetics were evaluated using the pseudo-first order Equation (3) and pseudo-second order Equation (4) models [54]:

(3) $$ln\left(q_{e}-q_{t}\right)={\ln q}_{e}-k_{1}t$$

(4) $$\frac{1}{q_{t}}=\frac{1}{k_{2}q_{e}^{2}}+\frac{t}{q_{e}}$$

where $k_{1}$ (h^−1^) is the pseudo-first-order rate constant, and $k_{2}$ (g·mg^−1^·h^−1^) is the pseudo-second-order rate constant. Linear fitting plots for both models for phosphate adsorption onto ABC-M700 are presented in the Supplementary Materials (Figure S3).

To further investigate the diffusion mechanisms, the intraparticle diffusion model Equation (5), the liquid film diffusion model Equation (6), and the particle diffusion model Equation (7) were applied [55].

(5) $$q_{t}=k_{t\;}t^{1/2\;}+A$$

(6) $$\ln\left(1-\frac{q_{t}}{q_{e}}\right)=\frac{D_{f}c_{s}}{h\;r\;c_{z}}t$$

(7) $$-\ln\left(1-{\left(\frac{q_{t}}{q_{e}}\right)}^{2}\right)=\frac{2\pi^{2}D_{p}}{r^{2}}t$$

where k_t_ (mg·g^−1^·h^−1/2^) is the intraparticle diffusion rate constant and A (mg·g^−1^) represents the boundary layer thickness; D_f_ and D_p_ are the diffusion coefficients (m^2^·h^−1^); $c_{s}$ (mg·L^−1^) and $c_{z}$ (mg·kg^−1^) denote the phosphate concentrations in solution and biochar (ABC-M700), respectively; r is the particle radius (3.7 × 10^−5^ m); t is the contact time (h); and h is the film thickness, assumed to be 1 × 10^−5^ m in poorly stirred systems. The linear fitting of the intraparticle diffusion model for phosphate adsorption onto ABC-M700 is presented in the Supplementary Materials (Figure S4), indicating a multilinear behavior that suggests multiple rate-limiting diffusion steps. Linear fittings of the film diffusion and particle diffusion models are also shown in Figure S5 to describe the transport mechanisms involved.

The pseudo-first-order model provided a poor fit with R^2^ = 0.87 and a maximum adsorption capacity (qₑ) of 3.56 mg PO_4_^3−^∙g^−1^, suggesting that physical adsorption mechanisms (e.g., van der Waals forces) play a minor role. In contrast, the pseudo-second-order model yielded an excellent fit (R^2^ = 1.00) with qₑ = 6.29 mg PO_4_^3−^∙g^−1^, confirming that chemisorption is the dominant mechanism, driven by strong covalent interactions between phosphate ions and metal (oxy)hydroxide functional groups (–Fe–OH, –Zn–OH, –Mn–OH). The high rate constant (k_2_ = 31.73 h^−1^) reflects the abundance of active sites generated after chemical modification, which facilitates the chemisorption process primarily through ligand exchange reactions.

Furthermore, the intraparticle diffusion model revealed two distinct linear regions: an initial rapid surface adsorption (R^2^ = 0.95), attributed to phosphate binding at readily accessible metal sites on ABC-M700, followed by a slower pore diffusion phase (R^2^ = 0.92) governed by phosphate migration into the mesopores of ABC-M700. The moderate fit of the film diffusion model (R^2^ = 0.87) suggests that while the transport of phosphate across the liquid film surrounding ABC-M700 particles contributes to initial adsorption, it is not the rate-limiting step [56]. This is typical for biochar with high surface reactivity, where chemical interactions reduce film resistance [57]. A better fit of the particle diffusion model (R^2^ = 0.93) further implies that intraparticle diffusion within the porous structure of ABC-M700 influences the later stages of adsorption. Nevertheless, the dominance of the pseudo-second-order kinetic model indicates that chemisorption at active metal sites is the primary mechanism, with pore diffusion playing a secondary role. The rapid kinetics observed for ABC-M700, reaching equilibrium within 30 min, are comparable to those reported for Mg-modified biochar (equilibrium within 35 min) [58] and are advantageous compared to other metal-modified biochar, such as Fe_12_LaO_19_@BC where equilibrium is attained within 60 min [59], zinc chloride-doped biochar (ZBC) from non-edible vegetal waste (equilibrium within 60 min) [60], and sawdust biochar treated with lime sludge (equilibrium within 10 h) [56]. Some metal-doped biochars, such as Fe–MnBC (Fe_3_O_4_ and MnO_2_), reach equilibrium at 4 h, whereas Fe/MnBC (MnFe_2_O_4_) requires 12 h [61]. However, other adsorbents, such as ZnO/betaine-modified biochar (ZnOBBNC), can achieve equilibrium within 10 min [62]. The synergy between the high surface area (33 m^2^·g^−1^) and metal-functionalized sites enables ABC-M700 to achieve both rapid kinetics and a high adsorption capacity, which are related to the phosphate adsorption mechanisms involved.

### 2.5. Phosphate Adsorption Isotherms

The equilibrium adsorption behavior of ABC-M700 for phosphate was assessed using the Langmuir and Freundlich isotherm models (Table 3). All values represent the best-fit parameters derived from triplicate-averaged data; standard deviations are not reported because model fitting was conducted using mean values only. The adsorption data were fitted to the Langmuir isotherm model, Equation (8), which assumes monolayer adsorption on a homogeneous surface with a finite number of identical sites, where saturation is achieved as the relative pressure approaches unity. The Freundlich isotherm model, Equation (9), which describes non-ideal adsorption on heterogeneous surfaces with varying energy binding sites, was also applied [63]:

(8) $$\frac{c_{e}}{q_{e}}=\frac{c_{e}}{q_{m}}+\frac{1}{k_{L}q_{m}}$$

(9) $$\ln q_{e}=\ln k_{F}+\frac{1}{n}ln{\;c}_{e}$$

In these equations, $q_{m}$ is the maximum adsorption capacity (mg·g^−1^), $k_{L}$ is the Langmuir constant related to adsorption affinity (L·mg^−1^), $k_{F}$ is the Freundlich constant indicative of adsorption capacity (mg·g^−1^ PO_4_^3−^), and 1/n reflects the intensity and heterogeneity of the adsorption process. Linear fitting of Langmuir and Freundlich isotherm models for phosphate adsorption onto ABC-M700 is presented in the Supplementary Materials (Figure S6), allowing comparison of each model’s suitability in representing equilibrium data.

According to the Langmuir model (R^2^ = 0.85), a maximum adsorption capacity (qₘ) of 73.22 mg·g^−1^ was determined, indicating that monolayer chemisorption predominates. In parallel, the Freundlich model (R^2^ = 0.82) indicated favorable adsorption on heterogeneous surfaces, as evidenced by an adsorption intensity parameter (1/n) of 0.28 (<1). These results suggest a hybrid adsorption mechanism involving monolayer coverage at high-affinity sites and multilayer adsorption on energetically diverse surfaces. This hybrid behavior implies that ABC-M700 can efficiently adsorb phosphate across a wide concentration range (5–2000 mg·L^−1^), making it suitable for both dilute wastewater and nutrient-rich agricultural runoff.

At neutral pH (pKa_2_ = 7.2), phosphate is present as H_2_PO_4_^−^ and HPO_4_^2−^, which favor strong ligand exchange through the formation of inner-sphere complexes via monodentate and bidentate interactions. Therefore, the sorption mechanism of the phosphate oxyanion is associated with the formation of complexes with hydroxyl groups on the hydrated metal (M: Fe, Zn, Mn) oxide layer impregnated within the biochar matrix (i.e., specific adsorption). Inner-sphere complexation is characterized by the replacement of surface hydroxyl groups with phosphate, forming stable bonds as described by Equation (10) [13].

(10) $$ABC-M700-OH+\begin{array}{c} H_{2}PO_{4}^{-}\\ \begin{array}{c} \begin{array}{c} HPO_{4}^{2-} \end{array} \end{array} \end{array}\begin{array}{c} \overset{Complexation}{\to }\\ pH\geq pH_{PZC} \end{array}\begin{array}{c} \begin{array}{c} \begin{array}{cc} \begin{array}{cc} ABC-M700-O-\begin{array}{c} \begin{array}{c} OH\\ | \end{array}\\ P\\ \begin{array}{c} \|\\ O \end{array} \end{array}-OH & +OH^{-} \end{array} & Monodentate \end{array}\\ \begin{array}{cc} \begin{array}{cc} ABC-M700<\begin{array}{c} O\\ O \end{array}>P⩽\begin{array}{c} OH\\ O \end{array} & +OH^{-} \end{array} & \begin{array}{c} Bidentate\\ Mononuclear \end{array} \end{array} \end{array}\\ \begin{array}{c} \begin{array}{cc} \begin{array}{cc} \begin{array}{c} ABC-M700-O\\ ABC-M700-O \end{array}>P⩽\begin{array}{c} OH\\ O \end{array} & +2OH^{-} \end{array} & \begin{array}{c} Bidentate\\ Binuclear \end{array} \end{array} \end{array} \end{array}$$

In addition, ion exchange reactions may also contribute to phosphate adsorption, as weakly bound anions (e.g., Cl^−^, OH^−^) at the metal sites are displaced by phosphate ions (H_2_PO_4_^−^) [64]. Furthermore, residual Ca^2+^ and Mg^2+^ in ABC-M700 (Table 1) can promote phosphate precipitation (e.g., Ca_3_(PO_4_)_2_) at alkaline pH [65], which may explain the adsorption behavior observed in this pH range despite the expected electrostatic repulsion. This precipitation mechanism will be further corroborated through fractionation assays. Additionally, at higher phosphate concentrations, surface precipitation may occur, leading to the formation of insoluble metal-phosphate phases (e.g., FePO_4_, Zn_3_(PO_4_)_2_) on the biochar surface [66], although these phases were not confirmed by XRD analysis.

**Figure 7:**
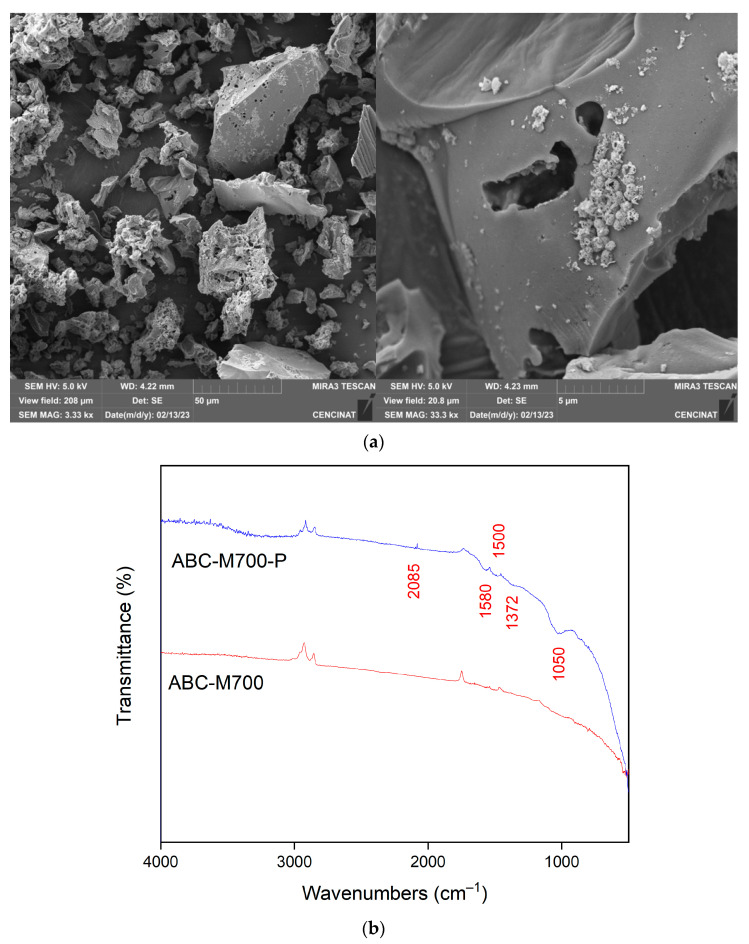
(**a**) SEM image at 50 µm and 5 µm scales and (**b**) FTIR spectrum of metal-modified annatto biochar saturated with phosphate (ABC-M700-P).

The SEM analysis performed after phosphate adsorption on ABC-M700-P (Figure 7a) revealed notable changes in surface morphology compared to the unloaded biochar. A visible surface coating or depositional layer was observed across the biochar surface, which was associated with phosphate binding and interactions with the supported metal species. These morphological features suggest that surface-level interactions occurred, consistent with the successful phosphate incorporation, as further confirmed by complementary FTIR analysis. The FTIR spectra of phosphate-loaded biochar (ABC-M700-P), compared to the pre-adsorption material (ABC-M700), exhibited distinct changes that confirm phosphate adsorption (Figure 7b). New absorption bands appeared at approximately 2085 cm^−1^, 1050 cm^−1^, 1372 cm^−1^, 1500 cm^−1^, and 1580 cm^−1^, which are either absent or significantly weaker in the spectrum of ABC-M700. After phosphate adsorption, the zone ranging from 3500 to 3050 cm^−1^ significantly decreased, indicating an interaction between the -OH groups and PO_4_^3−^. The pronounced band at 1050 cm^−1^ corresponds to the P-O stretching vibrations in orthophosphate groups, directly confirming the presence of adsorbed phosphate species [67]. Additional peaks at 1372 cm^−1^, 1500 cm^−1^, and 1580 cm^−1^ may be attributed to asymmetric and symmetric vibrations of phosphate complexes and their interactions with metal hydroxide groups on the biochar surface [68]. These spectral changes collectively support the conclusion that phosphate was successfully adsorbed onto the metal-modified biochar via inner-sphere complexation with Fe, Zn, and Mn (oxy)hydroxide functional groups. The findings agree with the desorption and fractionation results, offering additional spectroscopic evidence of metal–phosphate interactions occurring on the ABC-M700 surface following adsorption. Although post-adsorption elemental analysis was not performed in this study, we acknowledge its significance in providing a deeper understanding of phosphate binding mechanisms and the structural stability of the adsorbent. Additionally, the elemental composition of the unmodified biochar (ABC-N700) following water washing was not assessed, which could have provided valuable insights into the leachability of native elements. Such analysis would have clarified whether the observed compositional changes resulted from ion exchange reactions or simple dissolution processes. We recognize this as a limitation and highlight it as a key recommendation for future research. Nevertheless, based on previous studies and our earlier work employing the same metal-impregnation methodology, the variations in elemental composition are most likely attributable to a combination of ion exchange, surface complexation, and partial leaching phenomena. These considerations underscore the importance of conducting leaching stability assessments in future investigations to strengthen the mechanistic interpretation of phosphate adsorption.

### 2.6. Phosphate Fractionation

**Figure 8:**
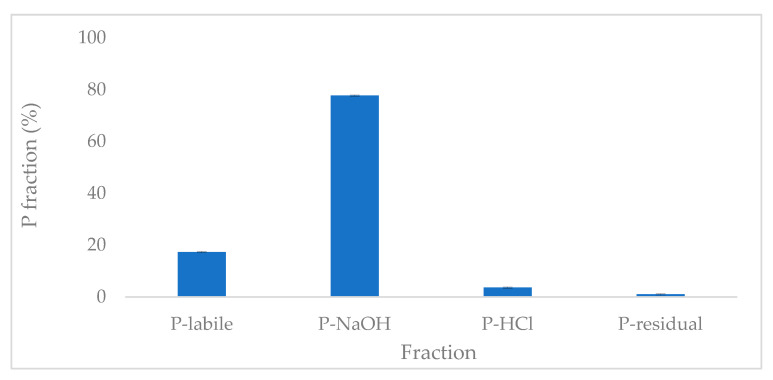
Phosphate fractionation of metal-modified annatto biochar saturated with phosphate (ABC-M700-P).

The fractionation of phosphorus adsorbed onto ABC-M700 revealed a distinct distribution across labile, NaOH–extractable (P–NaOH, which is associated with Fe/Al oxides), HCl–extractable (P–HCl, associated with Ca compounds), and residual fractions (Figure 8). The labile phosphorus fraction (17%) represents phosphate that is weakly retained via physical adsorption mechanisms such as electrostatic interactions and hydrogen bonding. This form is readily available for plant uptake, making it particularly valuable for agricultural applications requiring immediate nutrient availability. In contrast, the NaOH-extractable fraction (P–NaOH), which accounts for 78% of the total adsorbed phosphate, reflects strong chemisorption onto Fe, Zn, and Mn (oxy)hydroxides. This high proportion of metal-bound phosphate highlights the formation of stable inner-sphere complexes, thereby reducing the risk of phosphate leaching and enabling sustained nutrient retention. These findings align with previous studies on metal-modified biochar designed for long-term nutrient management during soil amendment. A smaller but relevant portion, 4%, was recovered in the HCl-extractable fraction (P–HCl), suggesting the presence of Ca-bound phosphate. This fraction is likely associated with interactions between phosphate and Ca^2+^ and Mg^2+^ species naturally present in annatto biochar, forming sparingly soluble precipitates (e.g., CaHPO_4_) with intermediate stability, thereby confirming a precipitation mechanism as part of the phosphate immobilization pathway. The residual fraction (1.0%) represents a negligible portion of irreversibly entrapped phosphate within the carbon matrix, reflecting the material’s efficiency in maximizing recoverable phosphorus. This multi–phase release profile, ranging from intermediate to sustained availability, positions ABC-M700 as a dual-functional material suitable for both wastewater treatment and precision agriculture. It supports soil fertility improvement over extended periods while addressing both environmental and agronomic objectives [15,69]. These results are consistent with prior fractionation studies conducted on metal-modified adsorbents, confirming that phosphate adsorption on ABC-M700 occurs predominantly via stable chemisorption pathways involving metal species [17,18].

The phosphate-loaded biochar (ABC-M700-P) was further characterized to assess the structural and functional changes after adsorption. While post-adsorption XRD analysis was not conducted, morphological and spectroscopic evaluations provided insight into phosphate retention mechanisms. SEM images revealed increased surface deposition on ABC-M700-P, and FTIR spectra displayed characteristic bands associated with phosphate-metal interactions, including a broad band near 1050 cm^−1^ attributed to P-O stretching vibrations. These changes, combined with the fractionation assay results showing that 78% of the retained phosphate was associated with metal-bound forms, confirm that phosphate adsorption was primarily facilitated by Fe, Zn, and Mn (oxy)hydroxide functional groups [17,18]. Although this study did not examine the individual effect of each metal through separate mono-metal modifications, the previous literature reports that Fe, Mn, and Zn exhibit higher affinity for phosphate via inner-sphere complexation [70,71]. Therefore, while the specific contribution of each metal cannot be isolated here, the available data suggest a synergistic effect among the incorporated metals in promoting phosphate retention. Further studies are planned to evaluate mono-metal modifications and better elucidate the role of each metal, thereby optimizing the formulation of ABC-M700.

### 2.7. Phosphate Desorption

**Figure 9:**
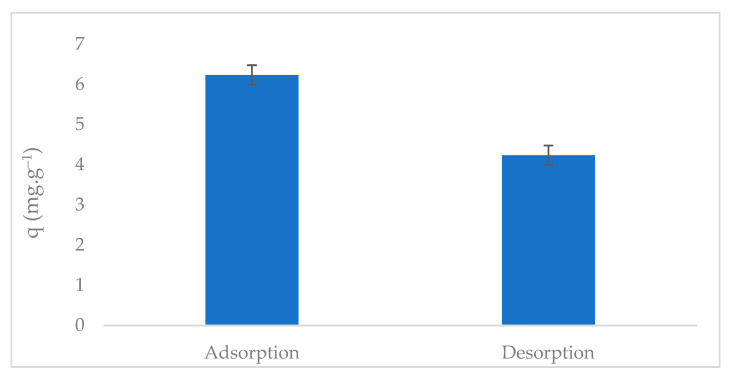
Phosphate adsorption capacity of metal-modified annatto biochar (ABC-M700) and desorption capacity from metal-modified annatto biochar saturated with phosphate (ABC-M700-P).

Desorption assays provide critical insights into the reversibility and stability of phosphate adsorbed onto metal-modified annatto biochar (ABC-M700). As shown in Figure 9, the adsorbed phosphate capacity in the first cycle was 6.29 mg·g^−1^, with a desorption efficiency of 68% corresponding to a desorption capacity of 4.28 mg·g^−1^ under 0.1 M NaHCO_3_ extraction. This behavior highlights the coexistence of labile (reversible) and stable (irreversible) phosphate retention mechanisms, which are consistent with the fractionation analysis discussed above.

The desorbed fraction (68%) primarily corresponds to weakly bound phosphate via electrostatic interactions or hydrogen bonding at the surface sites. Sodium bicarbonate (HCO_3_^−^) ions competitively displace these loosely held anions through competitive adsorption, enabling phosphate recovery for reuse. Conversely, the remaining phosphate content is attributed to strong chemisorption at the metal (oxy)hydroxide sites, where phosphate forms inner-sphere complexes or precipitates as insoluble phases. These bonds resist desorption under mild conditions, thereby ensuring the long-term retention of phosphorus. The desorbed fraction can be reused as fertilizer, thereby closing the phosphorus loop. In contrast, the retained phosphate is released slowly via ligand exchange or mineral dissolution, enhancing long-term soil fertility while mitigating the eutrophication risks associated with conventional fertilizer leaching. The desorption efficiency of ABC-M700-P (68%) exceeded that reported for other biochar using CaCl_2_ (60%) [72], although some materials exhibited phosphate desorption efficiencies above 92% when NaHCO_3_ was used [73]. However, NaHCO_3_ was proven inefficient for desorbing phosphate from Mg-biochar [74], and it underperforms relative to biochar dominated by physisorption, thereby underscoring the dominance of chemisorption in ABC-M700. Furthermore, several studies have explored alternative elution solutions for soil applications, as strongly basic or acidic solutions are often unsuitable for agricultural purposes.

### 2.8. Phosphate Removal Efficiency in Real Wastewater

ABC-M700 achieved 80% phosphate removal with an adsorption capacity of 34 mg·g^−1^ PO_4_^3−^), reducing the concentration from 82 mg·L^−1^ to 65 mg·L^−1^. However, this efficiency is lower than that observed in synthetic solution trials. The approximately 46% reduction in efficiency compared to synthetic solutions can be attributed to interference from competing ions such as sulfate (4% of removal with adsorption capacity 2.2 mg·g^−1^), nitrite (25% of removal with adsorption capacity 0.02 mg·g^−1^), fluoride (46% of removal with adsorption capacity 0.9 mg·g^−1^), cyanide (56% of removal with adsorption capacity 0.01 mg·g^−1^), ammonia (32% of removal with adsorption capacity 0.5 mg·g^−1^), total phosphorus (27% of removal with adsorption capacity 22.3 mg·g^−1^), and total nitrogen (25% of removal with adsorption capacity 2.9 mg·g^−1^), which compete with phosphate for adsorption sites. The application of metal-modified annatto biochar (ABC-M700) to real wastewater resulted in significant changes in key water quality parameters. Notably, the increase in pH can be attributed to the effect of metal (oxy)hydroxide functional groups, which generate hydroxide ions during deprotonation. In addition, both BOD_5_ and COD values increased in the treated water sample, suggesting that during the treatment process, some dissolved organic compounds may be released from the biochar or induce the transformation of complex organics into more oxidizable forms. Moreover, the concentrations of nitrates, bicarbonates, and chlorides increased relative to the initial composition of the wastewater. These changes imply that anion exchange processes occur during treatment, with native ions from the biochar being displaced and released into the effluent. In contrast, the levels of calcium and sodium decreased slightly, indicating ion adsorption, while potassium increased modestly, suggesting release. These shifts indicate differential ion exchange behavior, reflecting the varying affinities of these cations for the biochar surface. Furthermore, the concentrations of iron and manganese decreased in the final effluent, whereas zinc levels increased. Such variations suggest that the biochar preferentially adsorbs metal ions (e.g., iron and manganese) while releasing others, potentially owing to the formation of stable metal complexes or surface precipitation reactions during the treatment process.

Additionally, organic matter plays a fundamental role in the reduction of phosphate removal from real wastewater, as evidenced by the residual COD and surfactants (0.57 mg·L^−1^), which may form surface coatings on ABC-M700, blocking active sites. Total suspended solids TSSs (116 mg·L^−1^) can also adhere to the porous structure, reducing the available surface area for adsorption. Several complex effects have been observed during the treatment of real wastewater with ABC-M700. Consequently, it is essential to optimize the treatment conditions to ensure that phosphate removal is achieved without causing unintended alterations in overall water quality.

Treatment with ABC-M700 resulted in a substantial reduction in the total phosphorus and nitrogen contents, attributed to the adsorption of the organic fractions of both elements. Organic phosphorus, which comprises polyphosphates and organically bound phosphates, was effectively adsorbed onto the biochar matrix. Similarly, the organic nitrogen fraction includes nitrogen compounds that are less readily biodegradable. Consequently, a decrease in these nutrients (phosphorus and nitrogen) improves overall water quality, enhancing the potential for nutrient recovery and reuse in agricultural applications, thereby supporting sustainable wastewater management practices.

The comparative analysis presented in Table 4 indicates that the phosphate adsorption capacity of biochar is highly dependent on the precursor material, pyrolysis conditions, and surface modification. Reported adsorption capacities vary widely, from 3.7 mg·g^−1^ (mallee biochar) to 303.5 mg·g^−1^ (sewage sludge biochar). A comparison of the isotherm behavior of annatto biochar with other biochar derived from lignocellulosic biomass revealed its relative effectiveness. Studies have demonstrated that biochar derived from plant and wood residues often exhibits high adsorption capacities because of its microporous structures and surface functionalities [75].

Annatto biochar demonstrates competitive adsorption performance, particularly under real wastewater conditions. In contrast to many biochars that report high phosphate adsorption capacities under synthetic conditions, such as those derived from sugarcane leaves (81.8 mg·g^−1^), mallee (3.7 mg·g^−1^), *Mimosa pigra* trees (70.6 mg·g^−1^), and Fe_12_LaO_19_ spent coffee ground biochar (81.6 mg·g^−1^), annatto biochar was evaluated using real wastewater. In real wastewater, the presence of competing ions (e.g., sulfate, chloride, and carbonate) often reduces phosphate uptake efficiency. This observation highlights the robustness of annatto biochar in practical applications, as its adsorption capability was assessed in a complex matrix rather than under controlled, synthetic conditions. The distinction between synthetic and real wastewater environments further underscores the reliability of annatto biochar. It is noteworthy that biochars with high phosphate adsorption capacities are typically modified with metal compounds; however, some metals used for functionalization may not be recommended for phosphorus recycling due to potential soil contamination issues. Thus, metal-modified annatto biochar demonstrates practical applicability in real wastewater treatment, making it a promising candidate for large-scale phosphate recovery and water purification systems.

Biochars produced from agricultural residues, such as mallee (*Eucalyptus polybractea*) (3.7 mg·g^−1^), exhibit moderate adsorption capacities, with variations attributed to differences in ash content and inherent mineral composition. Conversely, *Mimosa pigra* tree (trunks) biochar modified with AlCl_3_ (70.6 mg·g^−1^) shows higher adsorption capacities, suggesting that the lignocellulosic structure and silica content of these materials contribute significantly to phosphate retention. Notably, sewage sludge biochar (303.5 mg·g^−1^) exhibited extremely high phosphate adsorption, a trend commonly observed in biochar derived from waste treatment residues. This high performance is attributed to the presence of inherent metal oxides (e.g., Fe, Al, and Ca compounds), which create additional active sites for phosphate binding through ligand exchange and electrostatic interactions. However, despite its high efficiency, sewage sludge biochar often presents challenges related to potential heavy metal contamination and regulatory restrictions on agricultural applications. Critically, annatto biochar overcomes some limitations associated with biochar from other sources because it is synthesized from uncontaminated, food-grade biomass generated within the same agro-industrial plant that processes annatto seeds. This closed-loop system enables the direct valorization of in-house waste into a high-performance adsorbent for treating the factory’s phosphate-rich wastewater, thereby eliminating the dependence on external treatment systems. Post-adsorption, the phosphate-saturated biochar (ABC-M700-P) can be safely applied to annatto agricultural fields, serving as a slow-release phosphorus fertilizer enriched with Fe, Zn, and Mn, essential micronutrients for crop growth. Such integration not only addresses wastewater pollution but also enhances soil fertility for annatto crop production, reducing dependency on synthetic fertilizers. Furthermore, the absence of toxic contaminants in annatto biochar ensures compliance with agricultural regulations and minimizes environmental risks. By aligning waste management, nutrient recovery, and agricultural productivity within a single operational framework, annatto biochar provides a scalable, economically viable model for sustainable agro-industries, prioritizing circularity, safety, and resource efficiency.

### 2.9. Practical Implications of Metal-Modified Annatto Biochar for Phosphate Adsorption

The application of metal-modified annatto biochar (ABC-M700) for phosphate removal significantly contributes to waste valorization, mitigating environmental impact, and promoting sustainable resource management. This study presents a promising, cost-effective, and environmentally sustainable alternative to conventional phosphate removal techniques for wastewater treatment, particularly in agricultural and industrial settings. Utilizing biochar derived from annatto processing residues, this approach aims to produce a functional adsorbent for the specialized treatment of wastewater effluent from industrial plants, thus generating phosphate-enriched material for agricultural use. This method aligns with circular economy principles by converting agricultural waste into high-value functional material.

Among the most relevant advantages of ABC-M700 is its high adsorption capacity when modified with transition metals, achieving up to 73.2 mg·g^−1^ PO_4_^3−^ in synthetic phosphate solutions. This performance surpasses that of conventional adsorbents such as activated carbon and other biochar, making ABC-M700 a highly effective material for phosphorus removal. Furthermore, the pH flexibility and versatility of ABC-M700 are significant advantages, as it exhibits stable phosphate removal across a broad pH range (3–9), unlike other phosphate adsorbents that require strict pH control. This ensures that ABC-M700 is applicable to diverse wastewater conditions without requiring expensive pH adjustments. Post-adsorption analyses revealed that 78% of the retained phosphate in ABC-M700 remained in stable metal-bound forms, facilitating its potential reuse as a slow-release phosphorus fertilizer for soil amendment, thereby supporting phosphorus recycling and sustainable agricultural practices.

In comparison to previous studies involving metal-based sorbents synthesized under idealized laboratory conditions, this study represents a significant advancement in real-world applicability. For instance, our earlier work (Guaya et al., 2022) involved doping Mn^2+^/Zn^2+^/Fe^3+^ oxy(hydroxide) nanoparticles onto a hydrotalcite (LDH) matrix synthesized from pure reagents, methods that, while effective, are costly and less scalable for industrial applications due to their complexity and reliance on controlled pH and multi-step processes [18].

In contrast, the current study utilizes low-cost annatto-derived biochar, a readily available material, as a support for in situ Fe, Zn, and Mn (oxy)hydroxide metal impregnation, thereby avoiding the use of synthetic precursors and harsh reaction conditions. Characterization techniques (BET, FTIR, XRD, and SEM) confirmed the successful incorporation of Fe, Zn, and Mn (oxy)hydroxide groups as functional groups and their role in enhancing phosphate adsorption (up to 73.22 mg·g^−1^). The results demonstrate the feasibility of a closed-loop valorization process, where an agro-industrial waste is transformed into a functional material with dual environmental benefits. The modified annatto-derived biochar (ABC-M700) exhibited a significant increase in phosphate adsorption capacity and favorable desorption behavior for nutrient recycling. This indicates its suitability not only for wastewater remediation but also for agronomic reuse. The simultaneous integration of Fe, Zn, and Mn, beneficial soil micronutrients, into the biochar matrix further enhances its value as a slow-release phosphate fertilizer. This demonstrates a clear improvement in environmental relevance and scalability over previous LDH-based materials. These findings suggest practical applicability at the industrial scale and reinforce the potential of ABC-M700 to replace more expensive or synthetically derived adsorbents. Although ABC-M700 offers significant advantages, challenges related to operational scalability and efficiency in complex wastewater matrices must be considered. Even though the system was validated in real wastewater matrices, confirming the functionality of the adsorbent in complex, non-ideal environments, the presence of sulfates and other anions, as well as organic matter in real wastewater, reduces the phosphate adsorption efficiency compared to synthetic solutions. Suspended solids and surfactants can adhere to the porous structure of biochar, partially blocking the active sites and reducing their surface area. These fouling and clogging phenomena necessitate additional pre-treatment steps, such as screening, coagulation, or filtration, to maintain adsorption efficiency over extended operational cycles.

The environmental compatibility of the selected metals (Fe, Zn, Mn), combined with the structural robustness and reusability of the biochar support, makes ABC-M700 a scalable, low-cost, and sustainable adsorbent. Its dual role in wastewater remediation and agronomic application reinforces its practical relevance for industries seeking circular economy solutions. This dual advantage further supports the use of ABC-M700 as an eco-friendly solution for closing the nutrient loop in agro-industrial applications.

Consequently, the implementation of regulatory measures and policies that promote agro-waste valorization and nutrient recovery incentives could encourage the adoption of ABC-M700 for sustainable phosphorus management, particularly in wastewater treatment facilities and the agricultural sector.

## 3. Materials and Methods

### 3.1. Annatto Residues Pretreatment and Chemicals

Annatto (*Bixa orellana*) seed waste was sourced from a local agro-industrial facility specializing in the processing of herbs and spices in Loja, Ecuador. The material was stored at room temperature until further use. The residue was pretreated by washing with 0.01 M NaOH, followed by drying at 100 °C for 24 h. Subsequently, the seed waste was oven-dried at 105 °C for an additional 24 h, ground, and sieved to a particle size of less than 2 mm before undergoing calcination. All reagents used for the chemical modification of biochar, including ferric chloride (FeCl_3_), zinc chloride (ZnCl_2_), and manganese chloride (MnCl_2_), were of analytical grade.

### 3.2. Biochar Production and Modification

Pyrolysis was performed in a muffle furnace at two target temperatures: 600 °C and 700 °C. The heating program consisted of an initial ramp rate of 3 °C·min^−1^ up to 100 °C, allowing for gradual moisture removal. At this point, ceramic crucible lids were placed to generate a low-oxygen atmosphere, simulating the inert conditions commonly used in biochar production. The temperature was then increased at a rate of 5 °C·min^−1^ until reaching 600 °C and 700 °C, which were maintained for 1 h to ensure complete carbonization. The resulting unmodified biochars were designated as ABC-N600 and ABC-N700, corresponding to their respective calcination temperatures.

Following calcination, the biochar was sieved using a 200-mesh screen. Biochar yield was calculated based on the weight loss before and after pyrolysis. For metal modification, a widely used aqueous impregnation method was adapted to deposit Fe^3+^, Zn^2+^, and Mn^2+^ (oxy)hydroxide species onto the biochar surface, without subsequent thermal treatment [17,18]. Specifically, 5 g of annatto-derived biochar was suspended in 25 mL of an aqueous solution containing 0.1 M of Fe(III), Zn(II), and Mn(II) salts. The pH was adjusted to 9, and the mixture was stirred continuously at 40 °C for 4 h. After impregnation, the material was thoroughly washed with deionized water to remove unbound ions and dried at 100 °C, yielding the modified biochar ABC-M600 and ABC-M700. Based on preliminary screening experiments for phosphate adsorption, the biochar modified at 700 °C (ABC-M700) demonstrated the highest adsorption capacity and was therefore selected for all subsequent characterization and adsorption–desorption studies. To simplify presentation and improve consistency throughout the manuscript, ABC-M700 is hereafter referred to as itself.

### 3.3. Characterization of Materials

The surface areas of both ABC-N700 and ABC-M700 were analyzed using nitrogen adsorption–desorption (BET single-point method). Gas flow was established at 35:65% N_2_:He, and measurements were performed with a Micrometrics Chemisorb 2720 (Micrometrics Chemisorb, Norcross, GA, USA). The elemental chemical compositions of the samples were determined by X-ray fluorescence using a Bruker S1 Titan 800 handheld analyzer (Bruker, Billerica, MA, USA). Surface morphology was examined by scanning electron microscopy (SEM) using a field-emission Tescan Mira 3 instrument (Brno, Czech Republic). Fourier-transform infrared (FTIR) spectroscopy was conducted to identify the functional groups present in the samples. FTIR spectra were recorded over the range of 4000–400 cm^−1^ using a FTIR spectrometer (Nicolet iS10, 4100 Jasco, Easton, MD, USA) operating at a frequency of 50–60 Hz. Additionally, the point of zero charge (PZC) of metal-modified biochar ABC-M700 was determined using the salt addition method. A series of NaCl solutions (0.01 and 0.05 M) with initial pH values ranging from 3 to 11 were prepared, and a known amount of biochar was added to each. After 24 h of agitation, the final pH was measured. The PZC was identified as the pH at which the net surface charge is zero, indicating optimal conditions for electrostatic adsorption processes.

### 3.4. Phosphorus Adsorption Experiments

The phosphate adsorption performance of ABC-M700 was evaluated in synthetic solutions at room temperature, and the average values are reported. Specifically, 0.1 g of ABC-M700 was equilibrated in 25 mL of phosphate solution with a concentration of 25 mg·L^−1^. Following filtration, the initial and final phosphate concentrations and equilibrium pH were measured by UV-Vis spectrophotometry at 420 nm, using the vanadomolybdophosphoric acid colorimetric method (4500-P C) [81]. The adsorption capacity (q_e_) was calculated using Equation (11).

(11) $$q_{e}=\frac{c_{i}-c_{f}}{m}\times V$$

where c_i_ and c_f_ are the initial and final phosphate concentrations (mg·L^−1^), V is the solution volume (L), and m is the adsorbent mass (g).

Adsorption was evaluated as a function of pH using phosphate solutions with pH values ranging from 3 to 11. Adsorption kinetics were assessed at pH 7 by sampling at specific time intervals (15 s to 24 h), and the remaining phosphate concentration was subsequently measured. The adsorption capacity at time t (q_t_) was determined using Equation (12).

(12) $$q_{t}=\frac{c_{i}-c_{t}}{m}\times V$$

where c_t_ is the phosphate concentration at time t (mg·L^−1^).

Phosphate adsorption isotherms were evaluated using initial phosphate concentrations ranging from 5 to 2000 mg·L^−1^ at pH 7, reflecting the natural pH of wastewater. For desorption and fractionation assays, 0.1 g of phosphate-saturated, modified annatto biochar (ABC-M700-P) was used. To determine the chemical forms of phosphate retained after adsorption, a sequential phosphate fractionation assay was performed. This method was adapted from Guaya et al. (2015) [52], based on the protocol developed by Hieltjes and Lijklema (1980) [82]. The fractionation experiment involved sequential extractions to isolate different phosphate-binding forms. First, 0.5 M ammonium chloride (NH_4_Cl, pH ~8.5) was used to extract the labile phosphate fraction weakly adsorbed to the biochar surface. This was followed by extraction with 0.1 M NaOH and subsequently 1 M NaCl to recover phosphate associated with Fe/Al (P–NaOH), representing the metal-bound fraction (e.g., iron, manganese, zinc). Phosphate associated with calcium Ca-bound P (P–HCl) was extracted using 1 M HCl extraction. Each extraction step was conducted under continuous agitation for 16 h at room temperature. The residual phosphate fraction was calculated by mass balance, as the difference between the total adsorbed phosphorus and the sum of extracted fractions. For desorption analysis, phosphate release from ABC-M700-P was evaluated using 20 mL of a 0.1 M sodium bicarbonate (NaHCO_3_) solution. All experiments were conducted in triplicate, and the data presented in figures and tables represent mean values with corresponding standard deviations.

### 3.5. Application of Metal-Modified Biochar ABC-M700 for Phosphate Adsorption in Real Wastewater from Annatto Residue Processing Plant

A real wastewater sample was collected from an annatto processing plant in Loja, Ecuador, operating under typical industrial conditions. Samples were collected from the outlet of a wastewater treatment plant (WWTP). Table 5 summarizes key physicochemical parameters, contextualizing the complexity of the wastewater and potential interferences in the phosphate adsorption process. Notably, the phosphate concentration (PO_4_^3−^) in the WWTP effluent was high (82 mg·L^−1^). Therefore, ABC-M700 was applied as a specific treatment to reduce phosphate content.

A batch adsorption test was conducted using 1 L of WWTP effluent (pH 7.05, PO_4_^3−^: 82 mg·L^−1^) treated with ABC-M700 at a dosage of 0.5 g·L^−1^. Treatment was performed at 20 °C with agitation at 150 rpm for 24 h, after which the final phosphate concentration and pH were measured. All analyses were conducted in an accredited laboratory to ensure accuracy and compliance with regulatory standards. The initial physicochemical characteristics of the untreated wastewater used in adsorption assays are summarized in Table S1 (Supplementary Materials). These baseline parameters were essential for evaluating the adsorption performance of ABC-M700 under real wastewater conditions. Analytical procedures for all measured parameters were conducted in accordance with the 23rd edition of the Standard Methods for the Examination of Water and Wastewater (October 2017) [81] and the 21st edition of the Official Methods of Analysis of the Association of Official Analytical Chemists (AOAC, 2019) [83].

## 4. Conclusions

This study presents a first-of-its-kind demonstration of valorizing agro-industrial annatto seed waste (*Bixa orellana*) to produce a functional biochar for the simultaneous removal of phosphate from real agro-industrial wastewater and its subsequent recovery for soil application. Annatto biochar (ABC-N700) was synthesized via pyrolysis at 700 °C and further enhanced by the successful deposition of ternary Fe, Zn, and Mn (oxy)hydroxide groups, yielding the metal-modified ABC-M700 material. Physicochemical characterization revealed a substantial increase in surface area from 4 m^2^·g^−1^ (ABC-N700) to 33 m^2^·g^−1^ (ABC-M700), while SEM and FTIR analyses confirmed the development of porous structures and the formation of metal-oxide/hydroxide phases. XRD analysis also revealed minor structural distortions attributable to metal impregnation.

The key innovation lies in the development of the ABC-M700 material, which exhibited a markedly improved phosphate adsorption capacity, more than 950% higher than its unmodified counterpart. The experimental results confirmed that phosphate adsorption was pH-dependent and followed the Langmuir isotherm model and pseudo-second-order kinetic model, reaching a maximum adsorption capacity of 73.22 mg·g^−1^ PO_4_^3−^. This enhancement is attributed to the presence of metal (oxy)hydroxide functional groups (e.g., Fe–OH, Zn–OH, Mn–OH), which facilitate phosphate retention through electrostatic attraction, ligand exchange, ion exchange, and surface precipitation mechanisms. Phosphate fractionation analysis revealed that 78% of the retained phosphate was present in stable metal-bound forms, and desorption studies confirmed that 68% of the adsorbed phosphate could be effectively recovered under the evaluated conditions. Although the specific contribution of each metal (Fe, Zn, Mn) was not individually assessed, the results suggest a synergistic interaction among the metal (oxy)hydroxide groups, a topic that will be explored in future studies. Importantly, validation under real wastewater conditions demonstrated that ABC-M700 achieved 80% phosphate removal despite the presence of competing ions, organic matter, and suspended solids. These findings set a precedent for integrating waste valorization into practical treatment solutions. The results highlight the feasibility of ABC-M700 not only for wastewater remediation but also as a slow-release phosphorus fertilizer, promoting a circular economy model that enables phosphorus recycling within the same agro-industrial process using locally available and environmentally safe resources. Unlike conventional materials synthesized from laboratory-grade chemicals, ABC-M700 biochar was produced using a scalable, low-cost method and modified with Fe, Zn, and Mn, micronutrients that support rather than harm soil health, avoiding energy-intensive steps. This research highlights the crucial role of circular economy principles in developing sustainable and integrated solutions to agro-industrial and environmental challenges.

## Figures and Tables

**Table 1:** Elemental composition of natural and modified annatto biochar.

Elements	ABC-N700 (%)			ABC-M700 (%)		
MgO	3.26	±	0.48	0.91	±	0.05
Al_2_O_3_	5.32	±	0.67	4.72	±	0.58
SiO_2_	4.24	±	0.27	3.07	±	0.26
P_2_O_5_	2.57	±	0.08	2.39	±	0.08
S	1.38	±	0.02	1.08	±	0.02
K_2_O	11.10	±	0.05	2.81	±	0.03
CaO	2.63	±	0.02	3.60	±	0.02
Cr_2_O_3_	ND			0.04	±	0.02
Fe_2_O_3_	0.14	±	0.00	0.52	±	0.00
Mn	0.04	±	0.00	0.24	±	0.01
Zn	0.03	±	0.00	0.71	±	0.01
Ba	0.01	±	0.04	0.02	±	0.08

ND: not detectable.

**Table 2:** Kinetic model parameters for phosphate adsorption onto metal-modified annatto biochar (ABC-M700).

Model	Kinetic Parameters	
Pseudo-first-order	q_e_ (mg∙g^−1^)	3.56
	k_1_ (h^−1^)	15.26
	R^2^	0.87
Pseudo-second-order	q_e_ (mg∙g^−1^)	6.29
	k_2_ (g·mg^−1^·h^−1^)	31.73
	R^2^	1.00
Intraparticular diffusion	k_t1_ (mg·g^−1^·h^−1/2^)	25.00
	R^2^	0.95
	k_t2_ (mg·g^−1^·h^−1/2^)	2.10
	R^2^	0.92
Film diffusion	D_f_ (m^2^∙h^−1^)	3.84 × 10^−6^
	R^2^	0.87
Particle diffusion	D_p_ (m^2^∙h^−1^)	6.45 × 10^−9^
	R^2^	0.93

**Table 3:** Isotherm model parameters of phosphate adsorption onto metal-modified annatto biochar (ABC-M700).

Langmuir		Freundlich	
q_m_ (mg∙g^−1^)	73.22	k_F_ (mg∙g^−1^)	6.41
k_L_ (L∙mg^−1^)	4.88 × 10^−3^	1/n	0.28
R^2^	0.85	R^2^	0.82

**Table 4:** Comparison of phosphate adsorption capacities of various biochar.

Biochar Source	Phosphate Adsorption Capacity (mg·g^−1^)/ Removal (%)	Water Type	Reference
Annatto biochar ABC-M700	73.2	Synthetic solution	This study
	34/80%	Real wastewater	
Mallee (*Eucalyptus polybractea*) biochar	3.7	Synthetic solution	[76]
Pine biochar Maiza-straw biochar	13.9 8.8	Synthetic solution	[77]
Anaerobically digested sugar beet tailings biochar	73%	Synthetic solution	[78]
Sewage sludge biochar	303.5	Synthetic solution	[79]
Sugarcane leaves Mg/Al-LDHs biochar	81.8 at pH 3	Synthetic solution	[64]
*Mimosa pigra* trees (trunks) modified with AlCl_3_ biochar	70.6	Synthetic solution	[80]
Fe_12_LaO_19_ spent coffee ground biochar	81.6 at 40 °C	Synthetic solution	[59]
Non-edible vegetable waste biochar modified with ZnCl_2_	47.8	Synthetic solution	[60]
Sawdust biomass treated with lime sludge biochar	16.7	Synthetic solution	[56]
Rice straw powder BC, Fe/MnBC (comprising Fe_3_O_4_ and MnO_2_), and Fe–MnBC (comprising MnFe_2_O_4_) biochar	Fe–MnBC: 135.9 Fe/MnBC: 17.9	Synthetic solution	[61]
ZnO/betaine-modified biochar adsorbent	256.5	Synthetic solution	[62]

**Table 5:** Physicochemical parameters of real wastewater collected from an annatto processing plant in Loja, Ecuador.

Parameter	Value (mg·L^−1^)
Phosphate (PO_4_^3−^)	82
Total phosphorus	41.2
QOD	63
BOD_5_	30
Nitrates	83.5
Nitrites	0.1
Total nitrogen	5.9
Sulfates (SO_4_^2−^)	28.2
Chloride (Cl^−^)	491.3
Oil and grease	157.7
pH	7.05
Temperature	21.4
* Surfactants	0.57
* Total suspended solids TSS	116

Source: Report on wastewater analysis by an accredited water laboratory. * Values reported by the factory.

## Data Availability

Data are contained within the article and Supplementary Materials.

## Supplementary Materials

The following supporting information can be downloaded at https://www.mdpi.com/article/10.3390/molecules30132842/s1, Figure S1. Visual comparison of raw annatto seed waste and the resulting ABC-M700 biochar after pyrolysis. Figure S2. Medusa diagram showing the aqueous speciation of Fe, Mn, and Zn at pH 9, supporting the formation of metal (oxy)hydroxides. Figure S3. Kinetic model fittings: (a) pseudo-first-order and (b) pseudo-second-order for phosphate adsorption on ABC-M700. Figure S4. Intraparticle diffusion model fitting indicates multi-step adsorption kinetics for ABC-M700. Figure S5. Diffusion model fittings: (a) film diffusion and (b) particle diffusion models for ABC-M700. Figure S6. Isotherm model fittings: (a) Langmuir and (b) Freundlich models for phosphate adsorption onto ABC-M700. Table S1. Physicochemical characteristics of untreated wastewater prior to phosphate adsorption experiments. Table S2. Physicochemical characteristics of wastewater after treatment with ABC-M700.

## References

[1] Koul B., Yakoob M., Shah M.P. (2022). Agricultural Waste Management Strategies for Environmental Sustainability. Environ. Res..

[2] Srivastava R.K., Shetti N.P., Reddy K.R., Nadagouda M.N., Badawi M., Bonilla-Petriciolet A., Aminabhavi T.M. (2023). Valorization of Biowastes for Clean Energy Production, Environmental Depollution and Soil Fertility. J. Environ. Manag..

[3] Masud M.A.A., Shin W.S., Sarker A., Septian A., Das K., Deepo D.M., Iqbal M.A., Islam A.R.M.T., Malafaia G. (2023). A Critical Review of Sustainable Application of Biochar for Green Remediation: Research Uncertainty and Future Directions. Sci. Total Environ..

[4] Jayakumar M., Hamda A.S., Abo L.D., Daba B.J., Prabhu S.V., Rangaraju M., Jabesa A., Periyasamy S., Suresh S., Baskar G. (2023). Comprehensive Review on Lignocellulosic Biomass Derived Biochar Production, Characterization, Utilization and Applications. Chemosphere.

[5] Novair S.B., Cheraghi M., Faramarzi F., Lajayer B.A., Senapathi V., Astatkie T., Price G.W. (2023). Reviewing the Role of Biochar in Paddy Soils: An Agricultural and Environmental Perspective. Ecotoxicol. Environ. Saf..

[6] Ginni G., Kavitha S., Kannah R.Y., Bhatia S.K., Kumar S.A., Rajkumar M., Kumar G., Pugazhendhi A., Chi N.T.L., Rajesh Banu J. (2021). Valorization of Agricultural Residues: Different Biorefinery Routes. J. Environ. Chem. Eng..

[7] Biswal B.K., Balasubramanian R. (2023). Use of Biochar as a Low-Cost Adsorbent for Removal of Heavy Metals from Water and Wastewater: A Review. J. Environ. Chem. Eng..

[8] Muddapur U.M., Turakani B., Jalal N.A., Ashgar S.S., Momenah A.M., Alshehri O.M., Mahnashi M.H., Shaikh I.A., Khan A.A., Dafalla S.E. (2023). Phytochemical Screening of Bixa Orellana and Preliminary Antidiabetic, Antibacterial, Antifibrinolytic, Anthelmintic, Antioxidant, and Cytotoxic Activity against Lung Cancer (A549) Cell Lines. J. King Saud. Univ. Sci..

[9] Pratibha G., Korwar G.R., Venkateswarlu B., Desai S., Chary G.R., Rao M.S., Srinivas K., Rao K.S., Rao C.H.S., Amalraj D.K.L.D. (2013). Utilization of Composted Bixa Shell with Different Bioinoculants as Soil Amendment for Ashwagandha and Bixa Growth. Ecol. Eng..

[10] Xiang W., Zhang X., Chen J., Zou W., He F., Hu X., Tsang D.C.W., Ok Y.S., Gao B. (2020). Biochar Technology in Wastewater Treatment: A Critical Review. Chemosphere.

[11] Otoni J.P., Matoso S.C.G., Pérez X.L.O., da Silva V.B. (2024). Potential for Agronomic and Environmental Use of Biochars Derived from Different Organic Waste. J. Clean. Prod..

[12] Silveira T.M.G., Tapia-Blácido D.R. (2018). Is Isolating Starch from the Residue of Annatto Pigment Extraction Feasible?. Food Hydrocoll..

[13] Guaya D., Hermassi M., Valderrama C., Farran A., Cortina J.L. (2016). Recovery of Ammonium and Phosphate from Treated Urban Wastewater by Using Potassium Clinoptilolite Impregnated Hydrated Metal Oxides as N-P-K Fertilizer. J. Environ. Chem. Eng..

[14] Guaya D., Valderrama C., Farran A., Sauras T., Cortina J.L. (2018). Valorisation of N and P from Waste Water by Using Natural Reactive Hybrid Sorbents: Nutrients (N,P,K) Release Evaluation in Amended Soils by Dynamic Experiments. Sci. Total Environ..

[15] Guaya D., Mendoza A., Valderrama C., Farran A., Sauras-Yera T., Cortina J.L. (2020). Use of Nutrient-Enriched Zeolite (NEZ) from Urban Wastewaters in Amended Soils: Evaluation of Plant Availability of Mineral Elements. Sci. Total Environ..

[16] Su J.-Z., Zhang M.-Y., Xu W.-H., Xu W.-M., Liu C., Rui S., Tuo Y.-F., He X.-H., Xiang P. (2024). Preparation and Applications of Iron/Biochar Composites in Remediation of Heavy Metal Contaminated Soils: Current Status and Further Perspectives. Environ. Technol. Innov..

[17] Guaya D., Maza L., Angamarca A., Mendoza E., García L., Valderrama C., Cortina J.L. (2022). Fe3+/Mn2+ (Oxy)Hydroxide Nanoparticles Loaded onto Muscovite/Zeolite Composites (Powder, Pellets and Monoliths): Phosphate Carriers from Urban Wastewater to Soil. Nanomaterials.

[18] Guaya D., Cobos H., Valderrama C., Cortina J.L. (2022). Effect of Mn2+/Zn2+/Fe3+ Oxy(Hydroxide) Nanoparticles Doping onto Mg-Al-LDH on the Phosphate Removal Capacity from Simulated Wastewater. Nanomaterials.

[19] Rosa D., Petruccelli V., Iacobbi M.C., Brasili E., Badiali C., Pasqua G., Di Palma L. (2024). Functionalized Biochar from Waste as a Slow-Release Nutrient Source: Application on Tomato Plants. Heliyon.

[20] Muhammad N., Ge L., Chan W.P., Khan A., Nafees M., Lisak G. (2022). Impacts of Pyrolysis Temperatures on Physicochemical and Structural Properties of Green Waste Derived Biochars for Adsorption of Potentially Toxic Elements. J. Environ. Manag..

[21] Xue L., Liu N., Zhang J., Sun Z., Fu S., Zhan X., Yang J., Zhou R., Zhang H., Liu H. (2023). Pyrolysis Temperature Had Effects on the Physicochemical Properties of Biochar. Plant Soil Environ..

[22] Song S., Liu S., Liu Y., Shi W., Ma H. (2024). Structural Characteristics and Adsorption of Phosphorus by Pineapple Leaf Biochar at Different Pyrolysis Temperatures. Agronomy.

[23] Hu X., Zhang R., Xia B., Ying R., Hu Z., Tao X., Yu H., Xiao F., Chu Q., Chen H. (2022). Effect of Pyrolysis Temperature on Removal Efficiency and Mechanisms of Hg(II), Cd(II), and Pb (II) by Maize Straw Biochar. Sustainability.

[24] Ambaye T.G., Vaccari M., van Hullebusch E.D., Amrane A., Rtimi S. (2021). Mechanisms and Adsorption Capacities of Biochar for the Removal of Organic and Inorganic Pollutants from Industrial Wastewater. Int. J. Environ. Sci. Technol..

[25] Eduah J.O., Nartey E.K., Abekoe M.K., Henriksen S.W., Andersen M.N. (2020). Mechanism of Orthophosphate (PO4-P) Adsorption onto Different Biochars. Environ. Technol. Innov..

[26] Li M., Liu J., Xu Y., Qian G. (2016). Phosphate Adsorption on Metal Oxides and Metal Hydroxides: A Comparative Review. Environ. Rev..

[27] Li R., Wang J.J., Gaston L.A., Zhou B., Li M., Xiao R., Wang Q., Zhang Z., Huang H., Liang W. (2018). An Overview of Carbothermal Synthesis of Metal–Biochar Composites for the Removal of Oxyanion Contaminants from Aqueous Solution. Carbon.

[28] Nosratabad N.A., Yan Q., Cai Z., Wan C. (2024). Exploring Nanomaterial-Modified Biochar for Environmental Remediation Applications. Heliyon.

[29] Wang S., Shan R., Wang Y., Lu L., Yuan H. (2019). Synthesis of Calcium Materials in Biochar Matrix as a Highly Stable Catalyst for Biodiesel Production. Renew Energy.

[30] Wang S., Kwak J.-H., Islam M.S., Naeth M.A., El-Din M.G., Chang S.X. (2020). Biochar Surface Complexation and Ni(II), Cu(II), and Cd(II) Adsorption in Aqueous Solutions Depend on Feedstock Type. Sci. Total Environ..

[31] Yankovych H., Novoseltseva V., Kovalenko O., Marcin Behunova D., Kanuchova M., Vaclavikova M., Melnyk I. (2021). New Perception of Zn(II) and Mn(II) Removal Mechanism on Sustainable Sunflower Biochar from Alkaline Batteries Contaminated Water. J. Environ. Manag..

[32] Tan W.-T., Zhou H., Tang S.-F., Chen Q., Zhou X., Liu X.-H., Zeng P., Gu J.-F., Liao B.-H. (2023). Simultaneous Alleviation of Cd Availability in Contaminated Soil and Accumulation in Rice (Oryza Sativa L.) by Fe-Mn Oxide-Modified Biochar. Sci. Total Environ..

[33] Xia H., Zhang Y., Chen Q., Liu R., Wang H. (2023). Unraveling Adsorption Characteristics and Removal Mechanism of Novel Zn/Fe-Bimetal-Loaded and Starch-Coated Corn Cobs Biochar for Pb(II) and Cd(II) in Wastewater. J. Mol. Liq..

[34] Díaz B., Sommer-Márquez A., Ordoñez P.E., Bastardo-González E., Ricaurte M., Navas-Cárdenas C. (2024). Synthesis Methods, Properties, and Modifications of Biochar-Based Materials for Wastewater Treatment: A Review. Resources.

[35] Adilina I.B., Widjaya R.R., Hidayati L.N., Supriadi E., Safaat M., Oemry F., Restiawaty E., Bindar Y., Parker S.F. (2021). Understanding the Surface Characteristics of Biochar and Its Catalytic Activity for the Hydrodeoxygenation of Guaiacol. Catalysts.

[36] Gale M., Nguyen T., Moreno M., Gilliard-AbdulAziz K.L. (2021). Physiochemical Properties of Biochar and Activated Carbon from Biomass Residue: Influence of Process Conditions to Adsorbent Properties. ACS Omega.

[37] Vaitkus A., Merkys A., Gražulis S. (2021). Validation of the Crystallography Open Database Using the Crystallographic Information Framework. J. Appl. Crystallogr..

[38] Profeta D.O., da Silva M.A., Faria D.N., Cipriano D.F., Freitas J.C.C., dos Santos F.S., Lima T.M., Vasconcelos S.C., Pietre M.K. (2025). Zeolite/Calcium Carbonate Composite for a Synergistic Adsorption of Cadmium in Aqueous Solution. Next Mater..

[39] Faria D.N., dos Santos F.S., Teixeira P.L.R., Cipriano D.F., Schettino M.A., de Pietre M.K., Freitas J.C.C. (2024). Synergistic Effect of CaCO3 Particles and Porous Carbon towards the Removal of Zn2+ Ions in Aqueous Solutions. Mater. Chem. Phys..

[40] Melo V.M.e., Ferreira G.F., Fregolente L.V. (2024). Sustainable Catalysts for Biodiesel Production: The Potential of CaO Supported on Sugarcane Bagasse Biochar. Renew. Sustain. Energy Rev..

[41] Chiang P.F., Zhang T.L., Giwa A.S., Maurice N.J., Claire M.J., Ali N., Shafique E., Vakili M. (2025). Effects of Calcium-Oxide-Modified Biochar on the Anaerobic Digestion of Vacuum Blackwater. Molecules.

[42] Ge L., Yao L., Wang Y., Zuo M., Liu Y., Wu K., Zhang W., Xu C. (2024). The Preparation, Layered Characterization and Potential Applications of Corncob Biochar. J. Anal. Appl. Pyrolysis.

[43] Wang X., Zhang P., Wang C., Jia H., Shang X., Tang J., Sun H. (2022). Metal-Rich Hyperaccumulator-Derived Biochar as an Efficient Persulfate Activator: Role of Intrinsic Metals (Fe, Mn and Zn) in Regulating Characteristics, Performance and Reaction Mechanisms. J. Hazard. Mater..

[44] Alahakoon Y.A., Wilson S.C., Peiris C., Ranasinghe Y.K., Gunatilake S.R., Zhang X., Mlsna T.E., Kumarasinghe U., Mohideen M.I.H., Gandra U.R. (2024). Carbothermally Synthesized, Lignin Biochar-Based, Embedded and Surface Deposited Nano Zero-Valent Iron Composites: Comparative Material Characterization, Selective Gas Adsorption and Nitroaromatics Remediation. Colloids Surf. C Environ. Asp..

[45] Wang Y., Lyu H., Du Y., Cheng Q., Liu Y., Ma J., Yang S., Lin H. (2024). Unraveling How Fe-Mn Modified Biochar Mitigates Sulfamonomethoxine in Soil Water: The Activated Biodegradation and Hydroxyl Radicals Formation. J. Hazard. Mater..

[46] Li M., Tang Y., Ren N., Zhang Z., Cao Y. (2018). Effect of Mineral Constituents on Temperature-Dependent Structural Characterization of Carbon Fractions in Sewage Sludge-Derived Biochar. J. Clean. Prod..

[47] Hasan M., Chakma S., Liang X., Sutradhar S., Kozinski J., Kang K. (2024). Engineered Biochar for Metal Recycling and Repurposed Applications. Energies.

[48] Wang Y., Wang K., Wang X., Zhao Q., Jiang J., Jiang M. (2024). Effect of Different Production Methods on Physicochemical Properties and Adsorption Capacities of Biochar from Sewage Sludge and Kitchen Waste: Mechanism and Correlation Analysis. J. Hazard. Mater..

[49] Liu X., Li G., Chen C., Zhang X., Zhou K., Long X. (2022). Banana Stem and Leaf Biochar as an Effective Adsorbent for Cadmium and Lead in Aqueous Solution. Sci. Rep..

[50] Zhou Q., Liao B., Lin L., Qiu W., Song Z. (2018). Adsorption of Cu(II) and Cd(II) from Aqueous Solutions by Ferromanganese Binary Oxide–Biochar Composites. Sci. Total Environ..

[51] Maneechakr P., Mongkollertlop S. (2020). Investigation on Adsorption Behaviors of Heavy Metal Ions (Cd^2+^, Cr^3+^, Hg^2+^ and Pb^2+^) through Low-Cost/Active Manganese Dioxide-Modified Magnetic Biochar Derived from Palm Kernel Cake Residue. J. Environ. Chem. Eng..

[52] Guaya D., Valderrama C., Farran A., Armijos C., Cortina J.L. (2015). Simultaneous Phosphate and Ammonium Removal from Aqueous Solution by a Hydrated Aluminum Oxide Modified Natural Zeolite. Chem. Eng. J..

[53] Guaya D., Valderrama C., Farran A., Cortina J.L. (2016). Modification of a Natural Zeolite with Fe(III) for Simultaneous Phosphate and Ammonium Removal from Aqueous Solutions. J. Chem. Technol. Biotechnol..

[54] Moraes C.S., Carneiro P.A., Faria D.N., Cipriano D.F., Freitas J.C.C., Amorim R.G., da Silva R.S., Pietre M.K. (2024). High Efficiency of Myclobutanil Adsorption by CTAB-Zeolite Structures: Experimental Evidence Meets Theoretical Investigation. Silicon.

[55] Wang J., Guo X. (2020). Adsorption Kinetic Models: Physical Meanings, Applications, and Solving Methods. J. Hazard. Mater..

[56] Yang S., Katuwal S., Zheng W., Sharma B., Cooke R. (2021). Capture and Recover Dissolved Phosphorous from Aqueous Solutions by a Designer Biochar: Mechanism and Performance Insights. Chemosphere.

[57] Yao Y., Gao B., Chen J., Yang L. (2013). Engineered Biochar Reclaiming Phosphate from Aqueous Solutions: Mechanisms and Potential Application as a Slow-Release Fertilizer. Environ. Sci. Technol..

[58] Almanassra I.W., Mckay G., Kochkodan V., Ali Atieh M., Al-Ansari T. (2021). A State of the Art Review on Phosphate Removal from Water by Biochars. Chem. Eng. J..

[59] Akindolie M.S., Choi H.J. (2023). Fe12LaO19 Fabricated Biochar for Removal of Phosphorus in Water and Exploration of Its Adsorption Mechanism. J. Environ. Manag..

[60] Chanda R., Jahid T., Karmokar A., Hossain B., Moktadir M.D., Islam M.D.S., Aich N., Biswas B.K. (2025). Functionalized Biochar from Vegetable Waste for Phosphorus Removal from Aqueous Solution and Its Potential Use as a Slow-Release Fertilizer. Clean. Mater..

[61] Beiyuan J., Wu X., Ruan B., Chen Z., Liu J., Wang J., Li J., Xu W., Yuan W., Wang H. (2024). Highly Efficient Removal of Aqueous Phosphate via Iron-Manganese Fabricated Biochar: Performance and Mechanism. Chemosphere.

[62] Nakarmi A., Bourdo S.E., Ruhl L., Kanel S., Nadagouda M., Alla P.K., Pavel I., Viswanathan T. (2020). Benign Zinc Oxide Betaine-Modified Biochar Nanocomposites for Phosphate Removal from Aqueous Solutions. J. Environ. Manag..

[63] Teixeira R.S., Schmidt D.V.C., dos Santos F.S., Cipriano D.F., Faria D.N., Freitas J.C.C., Pietre M.K. (2024). Nanostructured Faujasites with Different Structural and Textural Properties for Adsorption of Cobalt and Nickel. Braz. J. Chem. Eng..

[64] Li R., Wang J.J., Zhou B., Awasthi M.K., Ali A., Zhang Z., Gaston L.A., Lahori A.H., Mahar A. (2016). Enhancing Phosphate Adsorption by Mg/Al Layered Double Hydroxide Functionalized Biochar with Different Mg/Al Ratios. Sci. Total Environ..

[65] Hermassi M., Valderrama C., Font O., Moreno N., Querol X., Batis N.H., Cortina J.L. (2020). Phosphate Recovery from Aqueous Solution by K-Zeolite Synthesized from Fly Ash for Subsequent Valorisation as Slow Release Fertilizer. Sci. Total Environ..

[66] Guaya D., Jiménez R., Sarango J., Valderrama C., Cortina J.L. (2021). Iron-Doped Natural Clays: Low-Cost Inorganic Adsorbents for Phosphate Recovering from Simulated Urban Treated Wastewater. J. Water Process Eng..

[67] Yang J., Zhang M., Wang H., Xue J., Lv Q., Pang G. (2021). Efficient Recovery of Phosphate from Aqueous Solution Using Biochar Derived from Co-Pyrolysis of Sewage Sludge with Eggshell. J. Environ. Chem. Eng..

[68] Shin H., Tiwari D., Kim D.-J. (2020). Phosphate Adsorption/Desorption Kinetics and P Bioavailability of Mg-Biochar from Ground Coffee Waste. J. Water Process Eng..

[69] Hermassi M., Guaya D., Gibert O., Valderrama C., Cortina J.L., Ohtake H., Tsuneda S. (2019). Valorisation of Nutrients in Wastewaters Using Reactive Inorganic Sorbents BT—Phosphorus Recovery and Recycling. Phosphorus Recovery and Recycling.

[70] Yang Q., Wang X., Luo W., Sun J., Xu Q., Chen F., Zhao J., Wang S., Yao F., Wang D. (2018). Effectiveness and Mechanisms of Phosphate Adsorption on Iron-Modified Biochars Derived from Waste Activated Sludge. Bioresour. Technol..

[71] Hu Z.-T., Wang X.-F., Xiang S., Ding Y., Zhao D.-Y., Hu M., Pan Z., Varjani S., Wong J.W.-C., Zhao J. (2022). Self-Cleaning MnZn Ferrite/Biochar Adsorbents for Effective Removal of Tetracycline. Sci. Total Environ..

[72] Sarkhot D.V., Ghezzehei T.A., Berhe A.A. (2013). Effectiveness of Biochar for Sorption of Ammonium and Phosphate from Dairy Effluent. J. Environ. Qual..

[73] Novais S.V., Zenero M.D.O., Barreto M.S.C., Montes C.R., Cerri C.E.P. (2018). Phosphorus Removal from Eutrophic Water Using Modified Biochar. Sci. Total Environ..

[74] Li R., Wang J.J., Zhou B., Awasthi M.K., Ali A., Zhang Z., Lahori A.H., Mahar A. (2016). Recovery of Phosphate from Aqueous Solution by Magnesium Oxide Decorated Magnetic Biochar and Its Potential as Phosphate-Based Fertilizer Substitute. Bioresour. Technol..

[75] Ahmad M., Rajapaksha A.U., Lim J.E., Zhang M., Bolan N., Mohan D., Vithanage M., Lee S.S., Ok Y.S. (2014). Biochar as a Sorbent for Contaminant Management in Soil and Water: A Review. Chemosphere.

[76] Zhang H., Chen C., Gray E.M., Boyd S.E., Yang H., Zhang D. (2016). Roles of Biochar in Improving Phosphorus Availability in Soils: A Phosphate Adsorbent and a Source of Available Phosphorus. Geoderma.

[77] Zhao S., Wang B., Gao Q., Gao Y., Liu S. (2017). Adsorption of Phosphorus by Different Biochars. Spectrosc. Lett..

[78] Yao Y., Gao B., Inyang M., Zimmerman A.R., Cao X., Pullammanappallil P., Yang L. (2011). Biochar Derived from Anaerobically Digested Sugar Beet Tailings: Characterization and Phosphate Removal Potential. Bioresour. Technol..

[79] Yin Q., Liu M., Ren H. (2019). Biochar Produced from the Co-Pyrolysis of Sewage Sludge and Walnut Shell for Ammonium and Phosphate Adsorption from Water. J. Environ. Manag..

[80] Tran T.C.P., Nguyen T.P., Nguyen X.C., Nguyen X.H., Nguyen T.A.H., Nguyen T.T.N., Vo T.Y.B., Nguyen T.H.G., Nguyen T.T.H., Vo T.D.H. (2022). Adsorptive Removal of Phosphate from Aqueous Solutions Using Low-Cost Modified Biochar-Packed Column: Effect of Operational Parameters and Kinetic Study. Chemosphere.

[81] APHA (2017) (1999). Standard Methods for the Examination of Water and Wastewater.

[82] Hieltjes A.H.M., Lijklema L. (1980). Fractionation of Inorganic Phosphates in Calcareous Sediments. J. Environ. Qual..

[83] AOAC (2019). Official Methods of Analysis of the Association of Official Analytical Chemists: Official Methods of Analysis of AOAC International.

