# Supplementary material for: Closed-Loop Valorization of Annatto Seed Waste into Biochar: A Sustainable Platform for Phosphorus Adsorption and Safe Nutrient Recycling in Agro-Industries
Source: Molecules. 2025 Jul 2;30(13):2842. doi: 10.3390/molecules30132842 (PMC12250773; doi:10.3390/molecules30132842)
Supplement: Supplementary file 1 [file molecules-30-02842-s001.zip › molecules-3712410-supplementary.pdf]

**Supplementary Materials**

*Article*

# Closed-Loop Valorization of Annatto Seed Waste into Biochar: A Sustainable Platform for Phosphorus Adsorption and Safe Nutrient Recycling in Agro-Industries

**Diana Guaya [1,*], Camilo Piedra [2] and Inmaculada Carmona [3]**

[1] Department of Chemistry, Universidad Técnica Particular de Loja, Loja 110107, Ecuador
[2] Chemical Engineering School, Universidad Técnica Particular de Loja, Loja 110107, Ecuador
[3] EcoSs_Lab, Department of Biological Sciences, Universidad Técnica Particular de Loja, Loja 110107, Ecuador
* Correspondence: deguaya@utpl.edu.ec

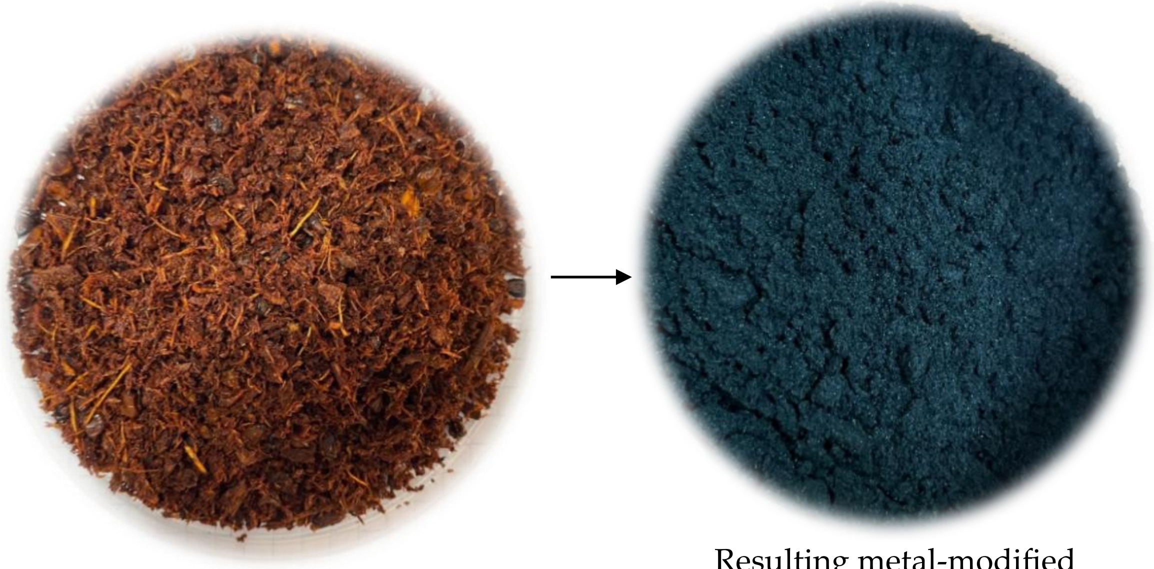


**Figure S1.** Visual comparison between raw annatto seed waste (left) and the resulting biochar after pyrolysis at 700 °C (right). The seed waste exhibits a reddish-orange coloration and fibrous texture characteristic of lignocellulosic agro-industrial residues, while the biochar displays a black, carbonized, and porous structure resulting from thermal decomposition. These photographs highlight the transformation and carbonization achieved during pyrolysis.

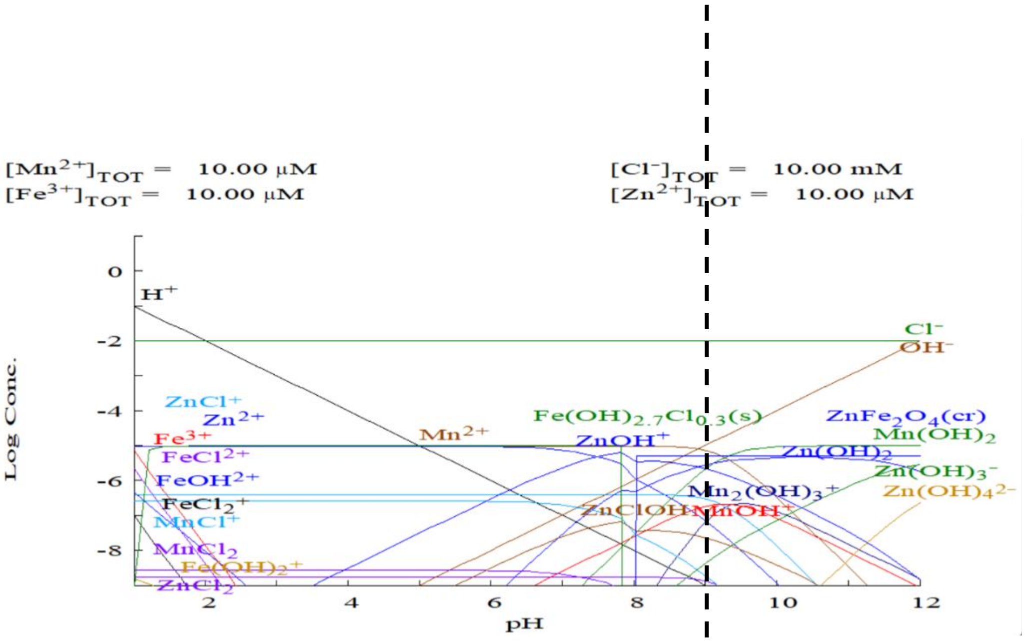


**pH= 9**

**Predominant aqueous species ($Fe^{3+}$, $Zn^{2+}$, $Mn^{2+}$) at experimental conditions**

**Figure S2.** Medusa diagram illustrating the pH-dependent aqueous speciation and precipitation domains of Fe, Mn, and Zn species at 25 °C. The diagram shows the formation of metal (oxy)hydroxides such as $Fe(OH)_3$, $Mn(OH)_2$, and $Zn(OH)_2$, supporting the expected conditions for their stabilization on ABC-M700 biochar.

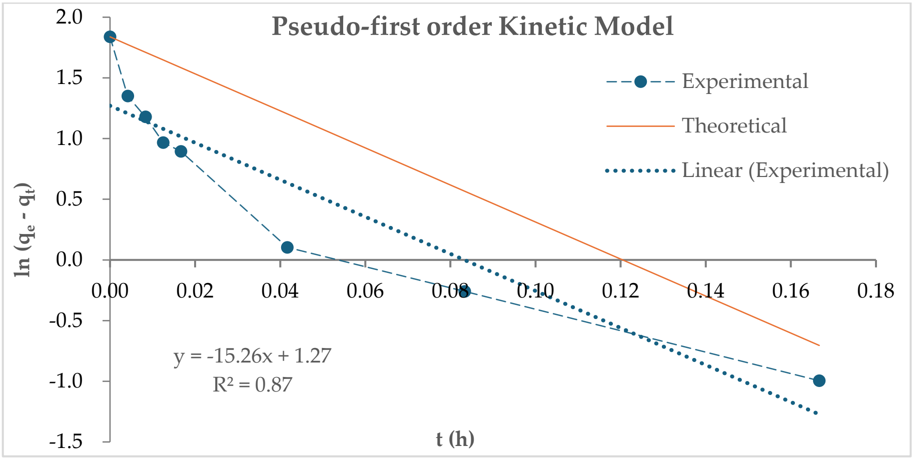


(a)

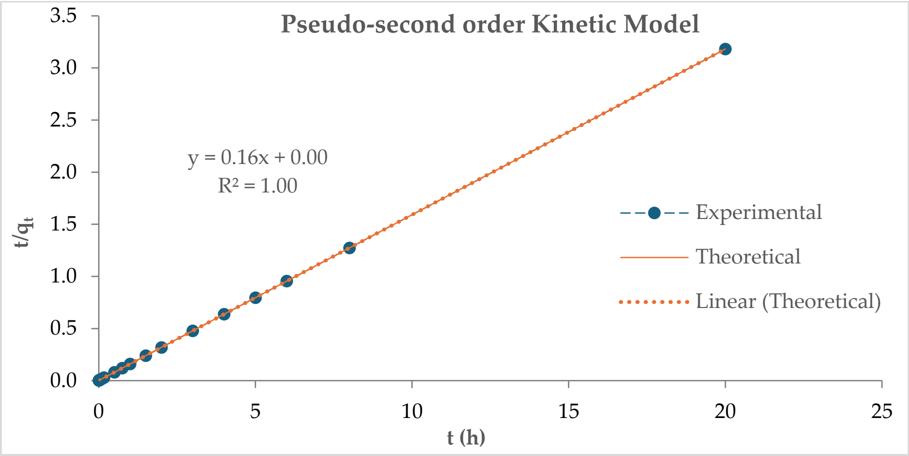


(b)

**Figure S3.** Linear fitting of (a) pseudo-first-order and (b) pseudo-second-order kinetic models for phosphate adsorption onto ABC-M700. The experimental data were obtained from triplicate experiments and fitted using their respective linear equations. $R^2$ values are indicated to assess the goodness of fit.

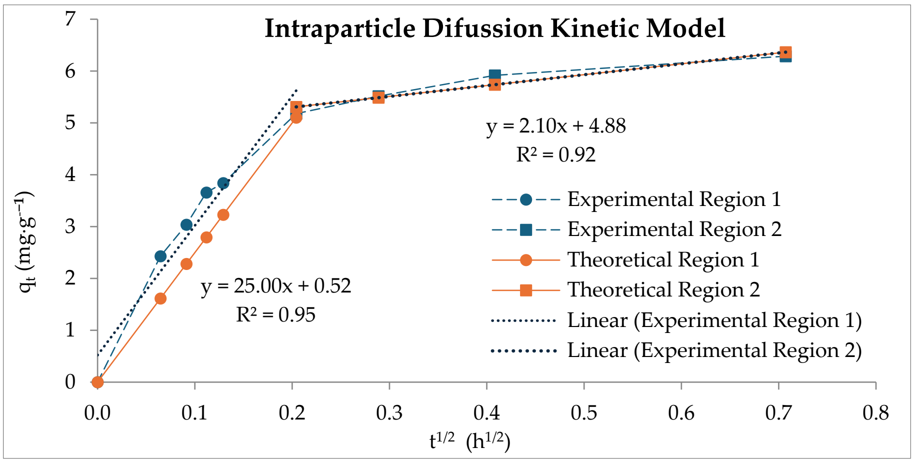


**Figure S4.** Linear fitting of the intraparticle diffusion model for phosphate adsorption onto ABC-M700. The experimental data were obtained from triplicate experiments and fitted using their respective linear equations. $R^2$ values are indicated to assess the goodness of fit. The multilinear trend suggests that adsorption is governed by multiple rate-limiting steps.

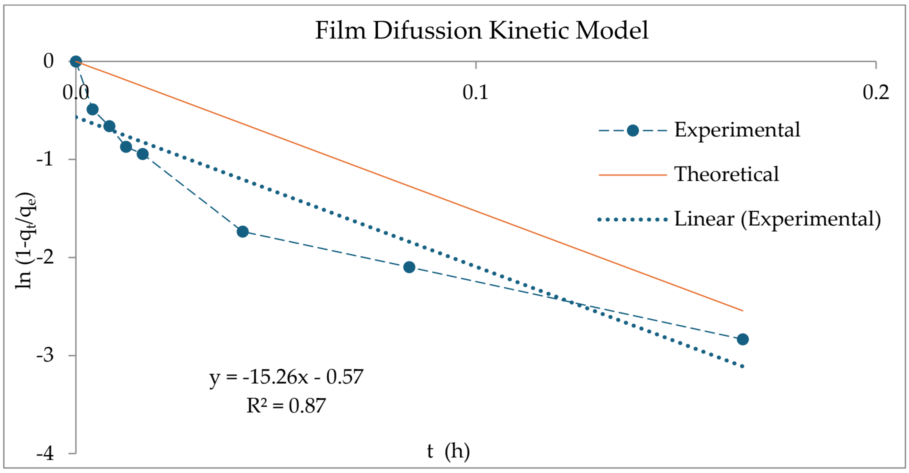


(a)

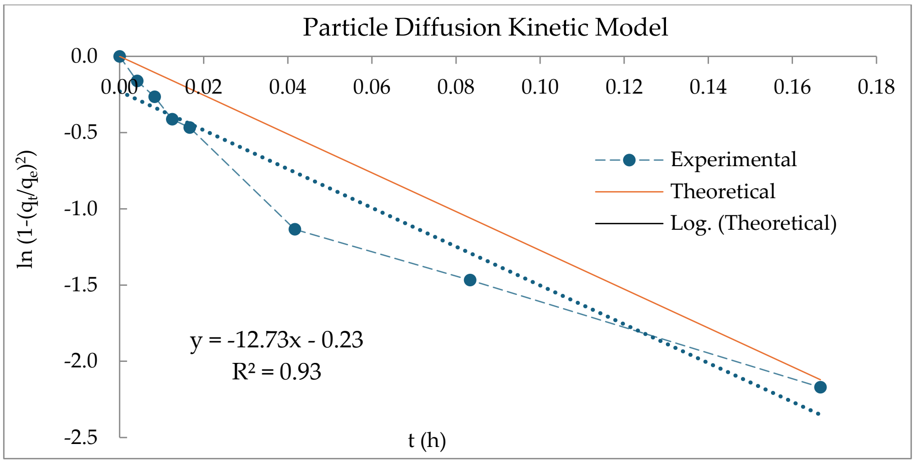


(b)

**Figure S5.** Linear fitting of the (a) film and (b) particle diffusion model applied to phosphate adsorption onto ABC-M700. The experimental data were obtained from triplicate experiments and fitted using their respective linear equations. $R^2$ values are indicated to assess the goodness of fit.

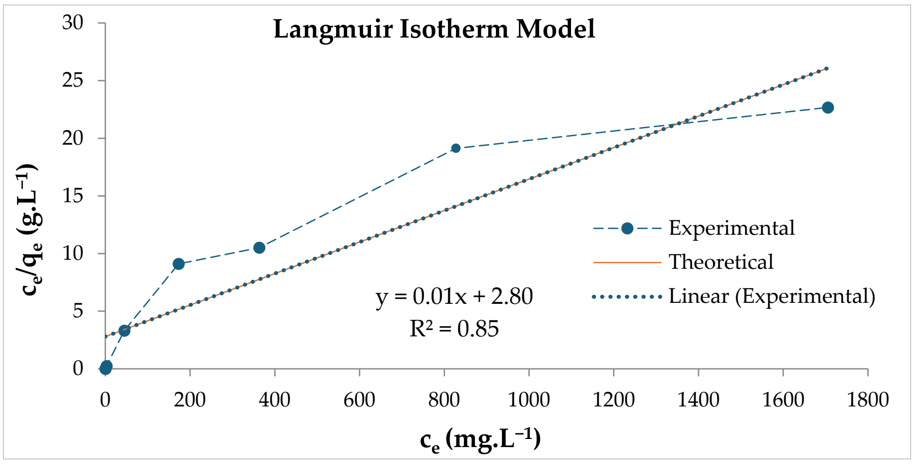


(a)

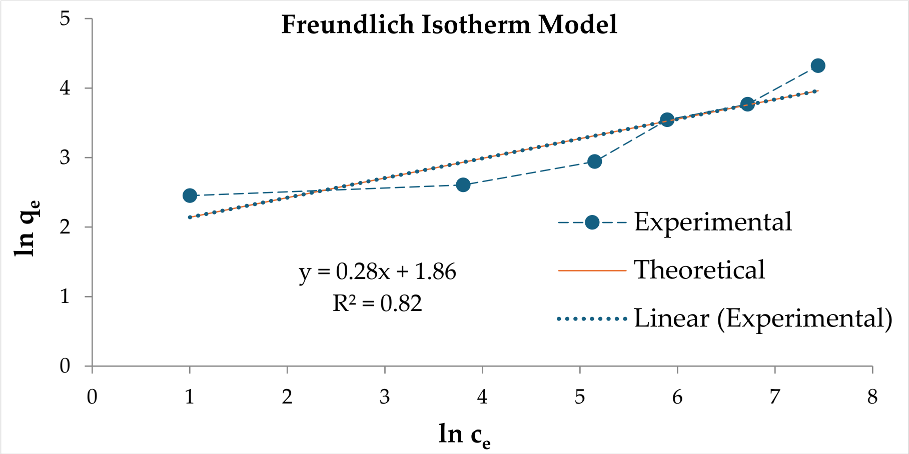


(b)

**Figure S6.** Linear fitting of (a) Langmuir and (b) Freundlich isotherm models for phosphate adsorption onto ABC-M700. The fitting was performed using the linearized equations for each model based on equilibrium adsorption data. Corresponding $R^2$ values are reported for model comparison.

**Table S1:** Results Report Record –Physicochemical characteristics of untreated wastewater prior to phosphate adsorption experiments.

**UNIVERSIDAD TÉCNICA PARTICULAR DE LOJA**

**REGISTRO DE INFORME DE RESULTADOS**

LABORATORIOS UTPL

*Laboratorio de Ensayo Acreditado por el SAE con acreditación N°.: SAE LEN 12-005*

**CODIGO:** R.7.8.2 **VERSION:** 5 **FECHA:** 2022-09-20 **ELABORADO POR:** Diego Maza Estrada **REVISADO Y APROBADO POR:** Diana Ines Hualpa

**Informe de Resultados Nro:** 2206181856

**Solicitud Nro:** 618 **Fecha del Informe:** 2022-11-21

**Sitio de análisis:** Laboratorios UTPL **Dirección:** San Cayetano Alto s/n, Loja.Ecuador

**Información Proporcionada por el Cliente:**

**Cliente:** Diana Guaya **Muestreador:** Diana Guaya

**Dirección:** Loja **Descripción:** Agua Residual

**Teléfono:** **Identificación:** Antes de Tratamiento

**Email:** deguaya@utpl.edu.ec **Fecha Toma Muestra:** 2022-11-09

**Información general de muestra recibida:**

**Fecha de recepción:** 2022-11-09

**Condiciones de recepción:** Las muestras son transportadas bajo cadena de frio, llegan al laboratorio a temperatura de (3 a 7) C°

**Resultados de análisis de muestra**

**Condiciones Ambientales durante el ensayo:** **Temperatura (°C):** 21.4 **Humedad (%)** 55

| Fecha de análisis | | Ítem de ensayo | Unidad | Resultado | Método de ensayo |
|---|---|---|---|---|---|
| Inicio | Fin | | | | |
| 2022-11-10 | 2022-11-10 | pH | - | 7.049 | AOAC, 973.41 |
| 2022-11-10 | 2022-11-10 | Sulfatos | mg/l | 28.2 | SM 4500-$SO_4$-E |
| 2022-11-10 | 2022-11-10 | Nitratos | mg/l | 83.53 | SM 4500-NO3-B |
| 2022-11-14 | 2022-11-14 | Cloruros | mg/l | 491.34 | SM 4500-Cl B |
| 2022-11-10 | 2022-11-10 | DQO | mg/l | 63 | SM 5220 D |
| 2022-11-10 | 2022-11-15 | DBO5 | mg/l | 30 | SM 5210 D |
| 2022-11-14 | 2022-11-14 | Fluoruro * | mg/l | 0.97 | SM 4500F-B,D |
| 2022-11-11 | 2022-11-11 | Cianuros * | mg/l | 0.009 | SM 4500-CN-E |
| 2022-11-15 | 2022-11-15 | Amonio * | mg/l | 0.79 | AOAC, 973.49 |
| 2022-11-10 | 2022-11-10 | Fosfatos * | mg/l | 82 | SM 4500-P-E. |
| 2022-11-11 | 2022-11-11 | Nitritos | mg/l | 0.054 | SM 4500 $NO_2$ - B |
| 2022-11-14 | 2022-11-14 | Bicarbonatos * | mg/l | 136.9 | SM 2320-B |
| 2022-11-17 | 2022-11-17 | Fósforo total * | mg/l | 41.19 | SM 4500-P-E. |
| 2022-11-17 | 2022-11-17 | Nitrógeno total * | mg/l | 5.88 | SM 4500-Norg - B |
| 2022-11-09 | 2022-11-10 | Coliformes Totales * | NMP/100ml | Ausencia | ISO 9308-2:2012 |
| 2022-11-09 | 2022-11-10 | Coliformes Fecales * | NMP/100ml | Ausencia | ISO 9308-2:2012 |
| 2022-11-14 | 2022-11-15 | Hierro | mg/l | 0.0846 | US EPA 3015; SM 3111 B |
| 2022-11-14 | 2022-11-15 | Manganeso | mg/l | 0.0581 | US EPA 3015; SM 3111 B |
| 2022-11-14 | 2022-11-15 | Zinc | mg/l | <0.05 (0.0342) | US EPA 3015; SM 3111 B |
| 2022-11-14 | 2022-11-15 | Calcio * | mg/l | 19.42 | US EPA 3015; SM 3111 A |
| 2022-11-14 | 2022-11-16 | Potasio * | mg/l | 64.8 | US EPA 3015; SM 3111 B |
| 2022-11-14 | 2022-11-17 | Sodio * | mg/l | 33.17 | US EPA 3015; SM 3111 A |

**Glosario:**

n/d: No disponible
<: Menor al límite de detección
% Sat OD: Porcentaje de saturación de oxigeno disuelto
UPtCo: Unidades de Platino Cobalto
NTU: Unidades nefelométricas de turbidez
uS/cm: microsiems por centímetro
NPM/100 ml: Número más probable de bacterias por 100 millitros
mg/l: miligramos por litro
IS: In Situ (En el sitio de muestreo)
SM: siglas en inglés de Método Estándar
AOAC: siglas en inglés de Asociación de Químicos Analíticos Oficiales



PBX:593-073701444 Ext. 3042 - 3041 E-mail: labutpl@utpl.edu.ec CP:11-01-608. San Cayetano Alto

**Observaciones:**

a) El informe de ensayo no se puede reproducir parcialmente, excepto en su totalidad con la aprobación escrita del laboratorio.
b) Los resultados representan exclusivamente la muestra (s) analizada (s).
c) Los ensayos marcados con (*) no están incluidos en el alcance de la acreditación del SAE
d) El laboratorio no se responsabiliza por la información proporcionada por el cliente (Registro R.4.4.1-B) que pueda afectar la validez de los resultados.
e) Cuando el resultado se expresa como <0,045 (0,016) significa que el límite más bajo de nuestra acreditación es 0,045 y el valor expresado entre paréntesis (0,016) corresponde a la concentración del parámetro en su muestra.

**Información Técnica:**

Los métodos de análisis para la determinación de cada uno de los parámetros, se basan en:
Edición 23th del Standard Methods, publicada en octubre de 2017.
Edición 21th del AOAC - Official Methods of Analysis. Association of Official Analytical Chemists, publicada en 2019.

| ELABORADO POR: | REVISADO Y APROBADO POR: |
| --- | --- |
| Ing. Cristian David Jumbo | Mgtr. Diego Ernesto Maza |
| **Técnico Analista** | **Líder Técnico** |

UNIVERSIDAD TÉCNICA PARTICULAR DE LOJA LABORATORIOS

----- Fin del Informe -----


PBX:593-073701444 Ext. 3042 - 3041 E-mail: labutpl@utpl.edu.ec CP:11-01-608. San Cayetano Alto

**Table S2:** Results Report Record – Physicochemical characteristics of wastewater after treatment with ABC-M700.

**UNIVERSIDAD TÉCNICA PARTICULAR DE LOJA**

**REGISTRO DE INFORME DE RESULTADOS**

LABORATORIOS UTPL

UTPL Laboratorios

*Laboratorio de Ensayo Acreditado por el SAE con acreditación N°.: SAE LEN 12-005*

**CODIGO:** R.7.8.2 **VERSION:** 5 **FECHA:** 2022-09-20 **ELABORADO POR:** Diego Maza Estrada **REVISADO Y APROBADO POR:** Diana Ines Hualpa

**Informe de Resultados Nro:** 2206181857

**Solicitud Nro:** 618 | **Fecha del Informe:** 2022-11-21

**Sitio de análisis:** Laboratorios UTPL | **Dirección:** San Cayetano Alto s/n, Loja.Ecuador

**Información Proporcionada por el Cliente:**

**Cliente:** Diana Guaya | **Muestreador:** Diana Guaya

**Dirección:** Loja | **Descripción:** Agua Residual

**Teléfono:** 3701444 | **Identificación:** Después de Tratamiento

**Email:** deguaya@utpl.edu.ec | **Fecha Toma Muestra:** 2022-11-09

**Información general de muestra recibida:**

**Fecha de recepción:** 2022-11-09

**Condiciones de recepción:** Las muestras son transportadas bajo cadena de frío, llegan al laboratorio a temperatura de (3 a 7) C°

**Resultados de análisis de muestra**

**Condiciones Ambientales durante el ensayo:** **Temperatura (°C):** 21.4 **Humedad (%)** 55

| Fecha de análisis | | Ítem de ensayo | Unidad | Resultado | Método de ensayo |
|---|---|---|---|---|---|
| Inicio | Fin | | | | |
| 2022-11-10 | 2022-11-10 | pH | - | 7.988 | AOAC, 973.41 |
| 2022-11-10 | 2022-11-10 | Sulfatos | mg/l | 27.09 | SM 4500-$SO_4$-E |
| 2022-11-10 | 2022-11-10 | Nitratos | mg/l | 119.55 | SM 4500-NO3-B |
| 2022-11-14 | 2022-11-14 | Cloruros | mg/l | 608.32 | SM 4500-Cl B |
| 2022-11-10 | 2022-11-10 | DQO | mg/l | 68 | SM 5220 D |
| 2022-11-10 | 2022-11-15 | DBO5 | mg/l | 32 | SM 5210 D |
| 2022-11-14 | 2022-11-14 | Fluoruro * | mg/l | 0.52 | SM 4500F-B,D |
| 2022-11-11 | 2022-11-11 | Cianuros * | mg/l | 0.004 | SM 4500-CN-E |
| 2022-11-15 | 2022-11-15 | Amonio * | mg/l | 0.54 | AOAC, 973.49 |
| 2022-11-10 | 2022-11-10 | Fosfatos * | mg/l | 65 | SM 4500-P-E. |
| 2022-11-11 | 2022-11-11 | Nitritos | mg/l | 0.042 | SM 4500 $NO_2$ - B |
| 2022-11-14 | 2022-11-14 | Bicarbonatos * | mg/l | 244.3 | SM 2320-B |
| 2022-11-17 | 2022-11-17 | Fósforo total * | mg/l | 30.04 | SM 4500-P-E. |
| 2022-11-17 | 2022-11-17 | Nitrógeno total * | mg/l | 4.41 | SM 4500-Norg - B |
| 2022-11-09 | 2022-11-10 | Coliformes Totales * | NMP/100ml | Ausencia | ISO 9308-2:2012 |
| 2022-11-09 | 2022-11-10 | Coliformes Fecales * | NMP/100ml | Ausencia | ISO 9308-2:2012 |
| 2022-11-14 | 2022-11-15 | Hierro | mg/l | <0.05 (0.0316) | US EPA 3015; SM 3111 B |
| 2022-11-14 | 2022-11-15 | Manganeso | mg/l | 0.4357 | US EPA 3015; SM 3111 B |
| 2022-11-14 | 2022-11-15 | Zinc | mg/l | <0.05 (0.0418) | US EPA 3015; SM 3111 B |
| 2022-11-14 | 2022-11-15 | Calcio * | mg/l | 17.56 | US EPA 3015; SM 3111 A |
| 2022-11-14 | 2022-11-16 | Potasio * | mg/l | 89.7 | US EPA 3015; SM 3111 B |
| 2022-11-14 | 2022-11-17 | Sodio * | mg/l | 28.68 | US EPA 3015; SM 3111 A |

**Glosario:**

n/d: No disponible
< : Menor al límite de detección
% Sat OD: Porcentaje de saturación de oxigeno disuelto
UPtCo: Unidades de Platino Cobalto
NTU: Unidades nefelométricas de turbidez
uS/cm: microsiems por centímetro
NPM/100 ml: Número más probable de bacterias por 100 mililitros
mg/l: miligramos por litro
IS: In Situ (En el sitio de muestreo)
SM: siglas en inglés de Método Estándar
AOAC: siglas en inglés de Asociación de Químicos Analíticos Oficiales



PBX:593-073701444 Ext. 3042 - 3041 E-mail: labutpl@utpl.edu.ec CP:11-01-608. San Cayetano Alto

**Observaciones:**

a) El informe de ensayo no se puede reproducir parcialmente, excepto en su totalidad con la aprobación escrita del laboratorio.
b) Los resultados representan exclusivamente la muestra (s) analizada (s).
c) Los ensayos marcados con (*) no están incluidos en el alcance de la acreditación del SAE
d) El laboratorio no se responsabiliza por la información proporcionada por el cliente (Registro R.4.4.1-B) que pueda afectar la validez de los resultados.
e) Cuando el resultado se expresa como <0,045 (0,016) significa que el límite más bajo de nuestra acreditación es 0,045 y el valor expresado entre paréntesis (0,016) corresponde a la concentración del parámetro en su muestra.

**Información Técnica:**

Los métodos de análisis para la determinación de cada uno de los parámetros, se basan en:
Edición 23th del Standard Methods, publicada en octubre de 2017.
Edición 21th del AOAC - Official Methods of Analysis. Association of Official Analytical Chemists, publicada en 2019.

**ELABORADO POR:**

Ing. Cristian David Jumbo
**Técnico Analista**

**REVISADO Y APROBADO POR:**

Mgtr. Diego Ernesto Maza
**Líder Técnico**

UNIVERSIDAD TÉCNICA PARTICULAR DE LOJA LABORATORIOS U.T.P.L.

------------------------------ Fin del Informe ------------------------------


PBX:593-073701444 Ext. 3042 - 3041 E-mail: labutpl@utpl.edu.ec CP:11-01-608. San Cayetano Alto
